# A Versatile Polyoxovanadate in Diverse Cation Matrices: A Supramolecular Perspective

**DOI:** 10.3389/fchem.2018.00469

**Published:** 2018-10-16

**Authors:** Srinivasa Rao Amanchi, Samar K. Das

**Affiliations:** School of Chemistry, University of Hyderabad, Central University, Hyderabad, India

**Keywords:** decavanadate cluster anion, diverse cations, crystal structures, supramolecular chemistry, decavanadate mineralogy

## Abstract

A series of decavanadate based compounds, formulated as [Co(H_2_O)_6_][{Na_4_(H_2_O)_14_}{V_10_O_28_}]·4H_2_O (**1**), [Zn(H_2_O)_6_][Na_3_(H_2_O)_14_] [HV_10_O_28_]·4H_2_O (**2**), [HMTAH]_2_ [{Zn(H_2_O)_4_}_2_{V_10_O_28_}]·2H_2_O (**3**), [{Co(3-amp)(H_2_O)_5_}]_2_ [3-ampH]_2_ [V_10_O_28_] · 6H_2_O (**4**), [4-ampH]_10_[{Na(H_2_O)_6_}{HV_10_O_28_}][V_10_O_28_]·15H_2_O (**5**), [{4-ampH}_6_ {Co(H_2_O)_6_}_3_][V_10_O_28_]_2_·14H_2_O (**6**), and [{4-ampH}_10_{Zn(H_2_O)_6_}][V_10_O_28_]_2_·10H_2_O (**7**), have been synthesized (where HMTAH = mono-protonated hexamethylenetetramine, 3-ampH = protonated 3-amino pyridine and 4-ampH= protonated 4-aminopyridine) from the relevant aqueous sodium-vanadate solution, by varying the pH of the solution and amino pyridine/hexamine derivatives as well as transition metal salts (Co(II)- and Zn(II)-salts). In this series of compounds **1**–**7**, the polyoxovanadate (POV) cluster [V_10_O_28_]^6−^ is the common cluster anion, stabilized by diverse cations. The diverse supramolecular patterns around the decavanadate cluster anion in different cationic matrices have been described to understand the microenvironment in the decavanadate-based minerals. All of these compounds have solvent water molecules in their respective crystal lattices. Since water can interact directly with cations and anions, providing an additional stability and structural diversity, we have analyzed supramolecular water structures in all these compounds to comprehend the role of the lattice water in the formation of natural decavanadate containing minerals. Compounds **1**–**7**, that are isolated at an ambient condition from aqueous solution, are characterized by routine spectral analysis, elemental analyses and finally unambiguously by single crystal X-ray crystallography.

## Introduction

**Figure 1:**
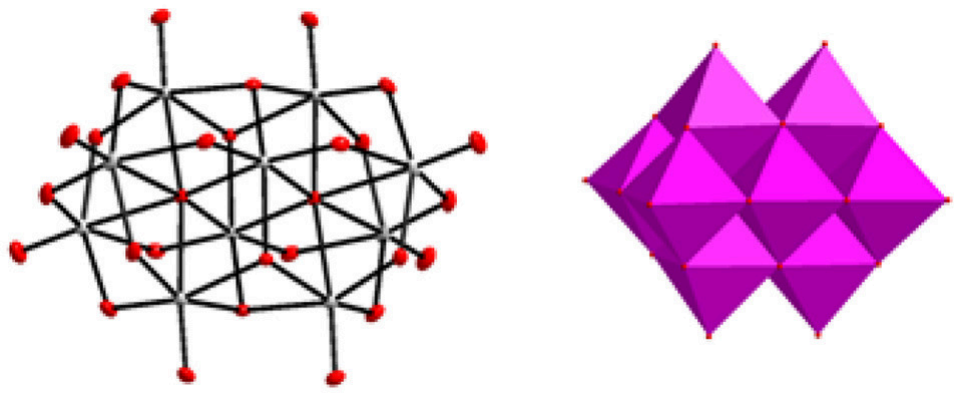
Decavanadate cluster [V_10_O_28_]^6−^ described in ball and stick and polyhedral representations respectively.

The modern chemical research on polyoxometalates (POMs)-based solid state materials fascinates synthetic chemists because of their potential applications in diverse research areas, such as, catalysis (Vazylyev et al., 2005; Hill, 2007; Zhou et al., 2014; Lechner et al., 2016; Mukhopadhyay et al., 2018), medicinal chemistry (Pope and Müller, 1991; Liu et al., 2012; Xie et al., 2018), and materials science (Guo et al., 2000; Rao et al., 2011; Kulikov and Meyer, 2013; Omwoma et al., 2015; Walsh et al., 2016). Among these, the area of polyoxovanadate (henceforth, POV) based materials have received special attention due to not only their diverse topologies (Miiller et al., 1990; Klemperer et al., 1992; Chen et al., 2005), structural (Miiller et al., 1987; Koene et al., 1999) and electronic properties (Müller et al., 1997) but also their fascinating versatile industrial applications, e.g., catalysis (Gao and Hua, 2006) and materials applications (Khan et al., 2003; Arumuganathan and Das, 2009; Chen et al., 2018). Among POVs, decavanadate cluster anion [V_10_O_28_]^6−^ is a versatile POM cluster anion, which is constructed by ten edge-shared VO_6_ octahedra with *D*_2*h*_ symmetry (Figure 1). Eight terminals-, fourteen doubly bridged (μ^2^)-, four triply bridged (μ^3^)- and two hexa bridged (μ^6^)-oxygen atoms exist within this [V_10_O_28_]^6−^ cluster anion. Numerious decavanadate based compounds are known in literature (Crans et al., 2017; Naslhajian et al., 2017; Yerra and Das, 2017) where the decavanadate cluster anion has been isolated using diverse cations including transition metal- and alkali metal-coordination complex cations; relevant supramolecular chemistry has also been described in the context of POV based materials chemistry (Sánchez-Lombardo et al., 2014; Wang et al., 2016). Choosing a particular cation plays a vital role in tuning the property of the resulting decavanadate cluster containing ion pair compound (Chatkon et al., 2013). The biological role of decavanadate cluster is enormous (Rehder, 2013; Winkler et al., 2017). Aureliano and co-workers described, in a perspective, the biological interactions of decavanadate with ion pump Ca^2+^-ATPase and compared the mode of action with those of already established ion-pump inhibitors of therapeutic importance (Aureliano et al., 2013). In addition to ion pumps, lipid structures also have been shown to represent biological targets for decavanadate (Aureliano and Crans, 2009). Crans and co-workers reported that rabbit skeletal muscle phosphorylase can be inhibited by decavanadate (Crans et al., 2004). They examined the interactions of decavanadate in an inorganic model system as well as in cells and determined the biological effects of decavanadate on rat basophilic leukemia (RBL-2H3) plasma membrane functions (Al-Qatati et al., 2013). In a recent report, de Carvalho and co-workers have demonstrated that decavanadate can interact with G-actin (multifunctional proteins), activating a protein conformational change and thereby that induces oxidation of the cysteine core residues (Marques et al., 2017). Even though, a good number of reports of the decavanadate cluster anion on diverse aspects of its biological significance is available including its catalytic applications (Kwon et al., 1988; Villa et al., 2001; Derat et al., 2006; Conte and Floris, 2010; Mestiri et al., 2013; Martín-Caballero et al., 2016; Amini et al., 2017; Huang et al., 2018), there are only few reports on decavanadate-based inorganic compounds along with their structural characterizations, that have been used as models to understand the formation of decavanadate-based minerals in Nature. D. C. Crans and C. C. McLauchlan and their co-workers have recently published an excellent review article covering the mineral-aspects of decavanadate compounds (Crans et al., 2017). There are more than 10 such minerals known, where the negative charges of decavanadate anion are counter-balanced mostly by alkali and alkali-earth metal cations and these minerals are stabilized with a good number of solvent water molecules. In order to comprehend their speciation in Nature, inorganic chemists have to synthesize decavanadate-based compounds with diverse cations from an aqueous solution and to characterize them crystallographically to investigate the micro-environments around decavanadate anion in these diverse cation matrices. This might offer an understanding the formation of decavanadate-based minerals in Nature. Moreover, exploration of the detailed interactions between decavanadate cluster anion and diverse counter cations is not only important for their categorization (da Silva et al., 2003) but also to gain the knowledge of new supramolecular chemistry of decavanadate ion (Crans et al., 1994; Yi et al., 2010; Chatkon et al., 2014). Thus we have synthesized and structurally characterized seven decavanadate based ion-pair compounds [Co(H_2_O)_6_][{Na_4_(H_2_O) _14_}{V_10_O_28_}] ·4H_2_O (**1**), [Zn(H_2_O)_6_] [Na_3_(H_2_O)_14_] [HV_10_O_28_] ·4H_2_O (**2**), [HMTAH]_2_[{Zn(H_2_O)_4_}_2_{V_10_O_28_}]·2H_2_O (**3**), [{Co(3-amp)(H_2_O)_5_]_2_[3-ampH]_2_[V_10_O_28_]·6H_2_O (**4**), [4-ampH]_10_[{Na(H_2_O)_6_} {HV_10_O_28_}][V_10_O_28_] ·15H_2_O (**5**), [{4-ampH}_6_{Co(H_2_O)_6_}_3_][V_10_O_28_]_2_·14H_2_O (**6**), and [{4-ampH}_10_{Zn(H_2_O)_6_}][V_10_O_28_]_2_ ·10H_2_O (**7**). Since in the present work, we are dealing with diverse cations ion-pairing with a common decavanadate polyanion, diverse supramolecular interactions are possible in their respective crystal structures. We have analyzed here the detailed supramolecular chemistry associated with each of these compounds and we have compared the microenvironment of decavanadate anion in these synthesized systems with that in some of the known decavanadate-based natural minerals (Graphical Abstract).

**Graphical Abstract:**
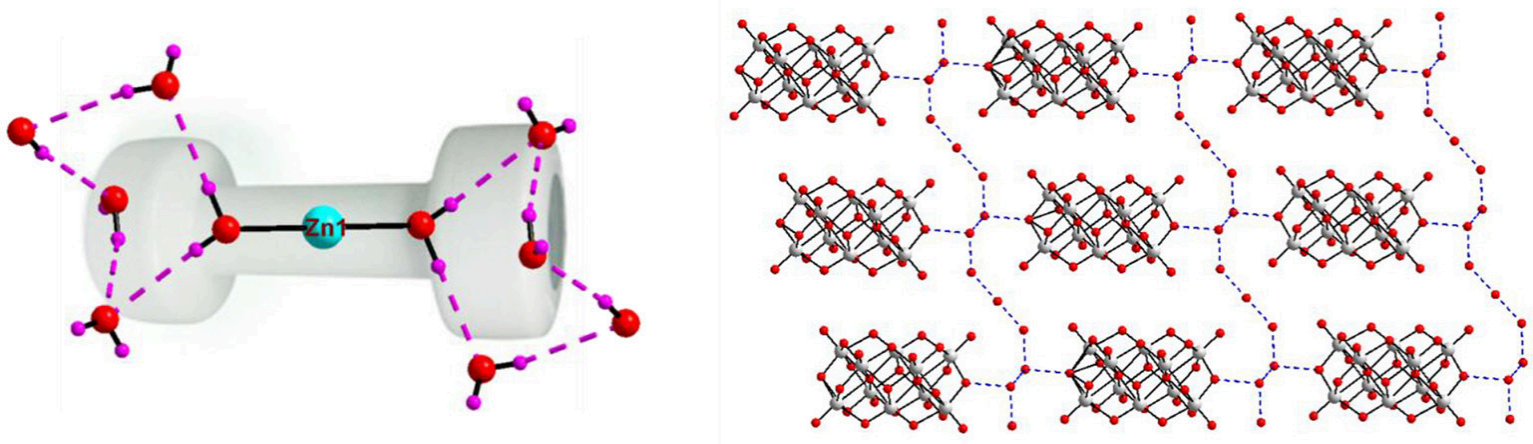
Decavanadate cluster {V_10_O_28_}^6−^ is the common anion to synthesize the materials **1–7**, ranging from discrete compounds to coordination polymers. Water clusters, such as, cyclic pentamers are established in some of the crystal structures due to water-water hydrogen bonding (O–H···O) interactions. There are analogies between these synthesized compounds and decavanadate based minerals in terms of the microenvironment around the isopolyanion.

## Experimental section

### Materials

Sodium metavanadate was received from SISCO Laboratory. The distilled water was used throughout the experiments. 2-Aminopyridine, 3-aminopyridine, 4-aminopyridine, and hexamine (hexamethylenetetramine) were received from CHEMLABS. Zn(NO_3_)_2_·6H_2_O and Co(NO_3_)_2_·6H_2_O were used as received from S.D. Fine and FINAR chemicals respectively.

### Physical measurements

Micro analytical (C, H, N) data were obtained with a FLASH EA 1112 Series CHNS Analyzer. Infrared (IR) spectra were recorded on KBr pellets with a JASCO FT/IR-5300 spectrometer in the region of 400–4000 cm^−1^.

### Preparation of compounds 1–7

#### Synthesis of [Co(H_2_O)_6_][{Na_4_(H_2_O)_14_}{V_10_O_28_}]·4H_2_O (1)

Sodium metavanadate (1 g, 4.13 mmol) was dissolved in 100 mL of water and its pH was adjusted to 9.0 by dil. HCl. In a separate beaker, the metal salt, Co(NO_3_)_2_·6H_2_O (0.5 g, 1.7 mmol) was dissolved in 20 mL of water. This reaction mixture of metal salt was added drop wise to the sodium vanadate solution with stirring. The resulting reaction mixture was stirred for 5 h (during stirring, the formation of precipitate / turbidity was dissolved by heating the reaction mixture at 70–80°C in three to four slots). The reaction mixture was then filtered and kept in open beaker for crystallization without any disturbance at room temperature. After 1 week, orange colored crystals formed, were filtered, washed with plenty of water and finally dried at room temperature. One of the single crystals, suitable for X-ray diffraction study, was selected and characterized structurally. Yield: 1.23 g. Anal. Calcd. (%) for CoH_48_Na_4_O_52_V_10_:_._ C, 0.00; H, 3.14, N, 0.00. Found: C, 0.03; H, 3.39, N, 0.04. IR (KBr pellet): (ν/cm^−1^) 3329, 3171, 1658, 1622, 1541, 1475, 1383, 1327, 1244, 1168, 991, 889, 829, 765, 617.

### Synthesis of [Zn(H_2_O)_6_][Na_3_(H_2_O)_14_][HV_10_O_28_]·4H_2_O (2)

The reaction procedure is same as the synthesis of compound **1** except Zn(NO_3_)_2_·6H_2_O (0.5 g, 1.68 mmol) was taken instead of Co(NO_3_)_2_·6H_2_O. The resulting reaction mixture was stirred for 5 h (during stirring, the formation of precipitate / turbidity was dissolved by heating the reaction mixture at 70–80°C in three to four slots). The reaction mixture was then filtered and kept in open beaker for crystallization without any disturbance at room temperature. After 1 week, orange colored crystals formed, were filtered, washed with plenty of water and finally dried at room temperature. One of the single crystals from, suitable for X-ray diffraction study, was selected and characterized structurally. Yield: 0.35 g. Anal. Calcd. (%) for H_58_Na_3_O_52_V_10_Zn: C, 0.00; H, 3.81; N, 0.00. Found: C, 0.09; H, 3.71; N 0.10. IR (KBr pellet): (ν/cm^−1^) 3337, 3229, 3057, 2085, 1614, 1560, 1485, 1398, 1327, 1332, 1280, 1072,922, 885, 829, 679, 584.

### Synthesis of [HMTAH]_2_[{Zn(H_2_O)_4_}_2_{V_10_O_28_}]·2H_2_O (3)

Sodium metavanadate (1 g, 4.13 mmol) was dissolved in 100 mL of water and its pH was adjusted to 3.0 by dil. HCl. In a separate beaker, the Zn(NO_3_)_2_·6H_2_O and hexamine (0.25 g, 1.68 mmol) were dissolved in 20 mL of water. This reaction mixture was added drop wise to the sodium vanadate solution with stirring. The resulting reaction mixture was stirred for 5 h (during stirring, the formation of precipitate / turbidity was dissolved by heating the reaction mixture at 70–80°C in three to four slots). The reaction mixture was then filtered and kept in open beaker for crystallization without any disturbance at room temperature. After 1 week, orange colored crystals formed, were filtered, washed with water and finally dried at room temperature. Yield: 0.27 g. Anal. Calcd. (%) for Zn_2_V_10_O_38_C_12_N_8_H_46_: C, 9.29; H, 2.99; N, 7.23. Found: C, 9.11; H, 2.78; N, 7.89. IR (KBr pellet): (ν/cm^−1^) 3420, 3310, 3190, 3078, 2918, 1647, 1614, 1570, 1464, 1386, 1313, 1230, 1182, 1086, 920, 883, 605.

### Synthesis of [{Co(3-amp)(H_2_O)_5_}]_2_[3-ampH]_2_[V_10_O_28_]·6H_2_O (4)

Sodium metavanadate (1 g, 4.13 mmol) was dissolved in 100 mL of water and its pH was adjusted to 3.0 by dil. HCl. In a separate beaker, the Co(NO_3_)_2_·6H_2_O (0.5 g, 1.7 mmol) and 3-aminopyridine (0.25 g, 1.68) were dissolved in 20 mL of water. This reaction mixture was added drop wise to the sodium vanadate solution with stirring. The resulting reaction mixture was stirred for 5 h (during stirring, the formation of precipitate/turbidity was dissolved by heating the reaction mixture at 70–80°C in three to four slots). The reaction mixture was then filtered and kept in open beaker for crystallization without any disturbance at room temperature. After 1 week, orange colored crystals formed, were filtered, washed with water and finally dried at room temperature. Yield: 0.27 g. Anal. Calcd. (%) for Co_2_V_10_O_44_C_20_N_8_H_58_: C, 13.79; H, 3.36; N, 6.43. Found: C, 13.21; H, 3.23; N, 6.89. IR (KBr Pellet): (ν/cm^−1^): 3456, 3322, 3170, 2998, 2978, 1654, 1625, 1567, 1469, 1398, 1267, 1156, 1076, 937, 896, 608.

### Synthesis of [4-ampH]_10_[{Na(H_2_O)_6_}{HV_10_O_28_}] [V_10_O_28_] ·15H_2_O (5)

Sodium metavanadate (1 g, 4.13 mmol) was dissolved in 100 mL of water and its pH was adjusted to 6.0 by dil. HCl. In a separate beaker, the 4-aminopyridine (0.25 g, 1.70 mmol) were dissolved in 20 mL of water. 4-aminopyridine solution was added drop wise to the sodium vanadate solution with stirring. The resulting reaction mixture was stirred for 5 h (during stirring, the formation of precipitate / turbidity was dissolved by heating the reaction mixture at 70–80°C in three to four slots). The reaction mixture was then filtered and kept in an open beaker for crystallization without any disturbance at room temperature. After 1 week, orange colored crystals formed, were filtered, washed with good amount of water and finally dried at room temperature. Yield: 0.47 g. Anal. Calcd (%) for V_20_NaO_77_C_50_N_20_H_113_: C, 18.37; H, 3.48; N, 8.57. Found: C, 18.15; H 3.99; N, 8.55. IR (KBr Pellet): (ν/cm^−1^): 3467, 3335, 3178, 2929, 1684, 1629, 1547, 1498, 1339, 1235, 1178, 1007, 967, 849, 619.

### Synthesis of [{4-ampH}_6_{Co(H_2_O)_6_}_3_][V_10_O_28_]_2_·14H_2_O (6)

Synthesis of compound **6** is same as that of compound **4** except 4-aminopyridine (0.25 g, 1.68 mmol) was taken (instead of 3-aminopyridine) in the reaction and maintained the same pH as was maintained in the synthesis of compound **4**. The resulting reaction mixture was stirred for 5 hrs (during stirring, the formation of precipitate / turbidity was dissolved by heating the reaction mixture at 70–80°C in three to four slots). The reaction mixture was then filtered and kept in open beaker for crystallization without any disturbance at room temperature. After 1 week, orange colored crystals formed, were filtered, washed with water and finally dried at room temperature. Yield: 1.87 g. Anal. Calcd. (%) for V_20_Co_3_O_88_C_30_N_12_H_106_: C,11.13; H, 3.30; N, 5.19. Found: C, 11.55; H, 3.79; N, 5.43. IR (KBr pellet) (ν/cm^−1^): 3427, 3345, 3196, 2822, 1690, 1645, 1556, 1489, 1338, 1268, 1189, 1039, 979, 890, 650.

### Synthesis of [{4-ampH}_10_{Zn(H_2_O)_6_}][V_10_O_28_]_2_ ·10H_2_O (7)

Synthesis of compound **7** is same as that of compound **4** except Zn(NO_3_)_2_·6H_2_O (0.5 g, 1.7 mmol) and 4-aminopyridine (0.25 g, 2.6 mmol) were taken (instead of Co(NO_3_)_2_·6H_2_O and 3-aminopyridine respectively) in the reaction and maintained the same pH as was maintained in the synthesis of compound **4**. The resulting reaction mixture was stirred for 5 h (during stirring, the formation of precipitate/turbidity was dissolved by heating the reaction mixture at 70–80°C in three to four slots). The reaction mixture was then filtered and kept in open beaker for crystallization without any disturbance at room temperature. After 1 week, orange colored crystals formed, were filtered, washed thoroughly with water and finally dried at room temperature. One of the single crystals, suitable for X-ray diffraction study, was selected and characterized structurally. The product obtained with Yield of 1.47 g. Anal. Calcd (%) for V_20_ZnO_72_C_50_N_20_H_102_: C, 18.65; H, 3.19; N, 8.70. Found: C, 18.32; H, 3.45; N, 8.63. IR (KBr pellet) (ν/cm^−1^): 3445, 3385, 3187, 2842, 1677, 1649, 1537, 1424, 1373, 1229, 1175, 1038, 929, 885, 643.

### X-ray data collection and structure determination

Data were measured on a Bruker SMART APEX CCD area detector system [λ(Mo Kα) = 0.71073 Å] with a graphite monochromator. 2400 frames were recorded with an ω scan width of 0.3°, each for 8 s keeping a crystal detector distance of 60 mm with a collimator of 0.5 mm. The data were reduced using SAINTPLUS program (software for the CCD detector system, Bruker Analytical X-ray Systems Inc., Madison, WI, 1998); the structures were solved using SHELXS-97 (Sheldrick, 1997) and refined using SHELXL-97 (Sheldrick, 1997). All non hydrogen atoms were refined anisotropically. We tried to locate the hydrogen atom of solvent water molecules for compound **2** through differential Fourier maps, but couldn't succeed. A summary of the crystallographic data and structure determination parameters are described in Table 1 for compounds **1–2**, in Table 2 for compounds **3**–**5** and in Table 3 for compounds **6** and **7**. Bond lengths and angles for decavanadate anionic cluster for **1** (as it is common cluster for all compounds) are provided in Table S1 they are in good agreement with those of reported decavanadate cluster anion [V_10_O_28_]^6−^ (Rao et al., 2011). CCDC- 1840294 (for compound **3**), - 1840295 (for compound **4**), - 1840296 (for compound **5**), - 1840297 (for compound **6**) and - 1840298 (for compound **7**) contain the supplementary crystallographic data for this paper. These data can be obtained free of charge from The Cambridge Crystallographic Data Center via www.ccdc.cam.ac.uk/ data_request/cif. CSD-434511 contains the supplementary crystallographic data for carbon free compound **2**; the details of the relevant crystal structure investigation may be obtained from the Fachinformationszentrum, Karlsruhe, D-76344 Eggenstein-Leopoldshafen, Germany (fax: (+49) 7247-808-666; e-mail: crysdata@fiz-karlsruhe.de) on quoting the depository number CSD-434511. Compound **1** (CSD 418030) is already structurally reported compound.

**Table 1:** Crystal data and structure refinement details for compounds **2**, **3**, and **4**.

**Entry**	**2**	**3**	**4**
Molecular formula	H_10_ZnNa_3_O_52_V_10_	C_12_H_24_N_8_O_38_V_10_Zn_2_	C_20_H_38_Co_2_N_8_O_44_V_10_
Formula weight	1485.42	1528.53	1721.84
Temperature (K)	298 (2)	298 (2)	298 (2)
Wavelength (Å)	0.71073	0.71073	0.71073
Crystal system	Triclinic	Monoclinic	Triclinic
Space group	*P*-1	*P21/c*	*P-1*
a (Å)	8.940 (3)	10.5993 (17)	10.5180 (16)
b (Å) 13.877 (4)	16.4355 (18)	11.9208 (18)	
c (Å)	18.360 (5)	13.865 (3)	12.711 (2)
α (deg)	91.766 (4)	90.00	97.818 (2)
β (deg) 91.744 (4)	120.191 (13)	107.937 (2)	
γ (deg)	104.705 (4)	90.00	100.240 (2)
Volume (Å^3^) 2200.4 (11)	2087.7 (6)	1460.8 (4)	
Z	2	2	1
ρ (g cm^−3^)	2.243	2.432	1.957
μ (mm^−1^) 2.718		3.378	2.181
F (000)	1438	1492	850
Crystal size (mm^3^)	0.24 × 0.18 × 0.14	0.36 × 0.18 × 0.14	0.46 × 0.34 × 0.20
θ range (°)	1.11 to 25.00	2.98 to 25.00	2.20 to 25.09
Reflections collected	18286	9787	13958
Unique reflections	6532	3683	5139
R(int)	0.0238	0.0819	0.0209
Parameters	7714/0 /640	3683/0/316	5139/0/455
Goodness of fit on F^2^	1.063	1.086	1.537
R_1_, *w*R_2_ [I > 2 sigma(I)]	0.0433, 0.1238	0.0885, 0.2660	0.0465,0.1582
R_1_, *w*R_2_ (all data)	0.0511, 0.1300	0.1387,0.2895	0.0479, 0.1598
Largest diff. Peak and			
hole (e.Å^−3^)	0.989/−1.051	1.854, −3.604	2.248, −0.840

**Table 2:** Crystal data and structure refinement details for compounds **5, 6**, and **7**.

**Entry**	**5**	**6**	**7**
Molecular formula	C_50_H_100_N_20_NaO_72_V_20_	C_30_H_88_Co_3_N_12_O_88_V_20_	C_50_H_96_N_20_O_72_V_20_Zn
Formula weight	3175.29	3220.71	3213.64
Temperature (K)	298 (2)	100 (2)	100 (2)
Wavelength (Å)	0.71073	0.71073	0.71073
Crystal system	Monoclinic	Triclinic	Monoclinic
Space group	*P21/c*	*P*-1	*P*2 (1)/c
a (Å)	13.1158 (10)	10.5396 (12)	13.087 (3)
b (Å)	20.0118 (16)	11.4041 (13)	19.997 (4)
c (Å)	20.1107 (16)	21.068 (2)	20.005 (4)
α(°)	90.000	99.843 (2)	90.000
β (°)	95.7520 (10)	91.015 (2)	95.680 (3)
γ(°)	90.000	91.547 (2)	90.000
Volume (Å^3^)	5251.9 (7)	2493.5 (5)	5209.6 (18)
Z	2	1	2
ρ (g cm^−3^)	2.008	2.145	2.049
μ (mm^−1^)	1.808	2.387	2.043
F (000)	3174	1597	3204
Crystal size (mm^3^)	0.36 × 0.24 × 0.18	0.34 × 0.18 × 0.16	0.26 × 0.20 × 0.18
θ range (°)	1.44 to 26.01	2.20 to 26.00	1.44 to 26.02
Reflections collected	54039	25672	50931
Unique reflections	10321	9682	10234
R(int)	0.0318	0.0230	0.0476
Parameters	10321/0/936	9682/0/898	10234/0/832
GOF on F^2^	1.025	1.188	1.147
R_1_, *w*R_2_[I > 2σ(I)]	0.0355, 0.0962	0.0465, 0.1044	0.0468, 0.1020
R_1_, *w*R_2_ (all data)	0.0406, 0.0997	0.0500, 0.1060	0.0549, 0.1056
Largest diff. Peak and hole (e.Å^−3^)	2.108, −0.417	2.084 and −1.072	0.551, −0.345

**Table 3:** Decavanadate-based minerals.

**Name of minerals**	**Formulas**	**Type of decavanadate**	**References**
Huemulite	Na_4_ Mg(V_10_O_28_) •24H_2_O	Oxidized	Colombo et al., 2011
Hughesite	Na_3_Al(V_10_O_28_) •22H_2_O	Oxidized	Rakovan et al., 2011
Hummerite	K_2_Mg_2_(V_10_O_28_) •16H_2_O	Oxidized	Hughes et al., 2002
Kokinosite	Na_2_Ca_2_(V_10_O_28_)•24H_2_O	Oxidized	Kampf et al., 2014
Lasalite	Na_2_Mg_2_ (V_10_O_28_) •20H_2_O	Oxidized	Hughes et al., 2008
Magnesiopascoite	MgCa_2_(V_10_O_28_) •16H_2_O	Oxidized	Kampf and Steele, 2008
Pascoite	Ca_3_(V_10_O_28_) •17H_2_O	Oxidized	Hughes et al., 2005
Postite	MgAl_2_(V_10_O_28_)(OH)_2_•27H_2_O	Oxidized	Kampf et al., 2012
Schindlerite	Na_2_(H_3_O)_4_(V_10_O_28_) •10H_2_O	Oxidized	Kampf et al., 2013b
Wernerbaurite	Ca_2_(H_3_O)_2_(V_10_O_28_) •16H_2_O	Oxidized	Kampf et al., 2013b
Gunterite	Na_4_(H_2_V_10_O_28_) •22H_2_O	Protonated	Kampf et al., 2011b
Rakovanite	Na_3_(H_3_V_10_O_28_) •15H_2_O	Protonated	Kampf et al., 2011a
Nashite	Na_3_Ca_2_[(V^IV^${\text{V}}_{9}^{\text{V}}$)O_28_] •24H_2_O	Mixed valent	Kampf et al., 2013a

## Results and discussion

### Synthesis

The synthetic method for the title compounds is simple one pot wet synthesis at a moderate temperature and their isolations are dependent on pH of the concerned solutions. Formation of the decavanadate cluster is feasible in the pH range of 2–9 in the solution, where pH of the solution is maintained by adding dil. HCl acid. Here, we have isolated seven ion pair compounds by altering the various cations and simultaneously pH condition. The formation of decavanadate cluster anion can be explained by protonation of vanadate anion and followed by a series of condensation reactions. The overall chemical reaction for the formation of decavanadate anion is given in equation 1. As expected, IR spectra of all these synthesized compounds reveal the presence of decavanadate anionic cluster as a common anionic component in title compounds. Vanadium-oxygen IR stretching frequencies of the decavanadate cluster anion containing ion pair compound depends on the type of the cation, associated with the anion.

(1) $$\begin{array}{l} 10{\text{VO}}_{3}^{-}+4{\text{H}}^{+}\to {\left[{\text{V}}_{10}{\text{O}}_{28}\right]}^{6-}+{2\text{H}}_{2}\text{O} \end{array}$$

Generaly, decavanadate cluster anion decomposes to tetravanadate above pH 7. We still could isolate compounds **1** and **2** at a higher pH value (more than pH 7). We believe that, in our synthesis, although the starting PH is in the range of 9-10, it drops down when the salts are added. That is why, we could isolate decavanadate from the reaction mixture.

### Molecular structures and supramolecular chemistry

#### Compound [Co(H_2_O)_6_][{Na_4_(H_2_O)_14_}{V_10_O_28_}]·4H_2_O (1)

**Figure 2:**
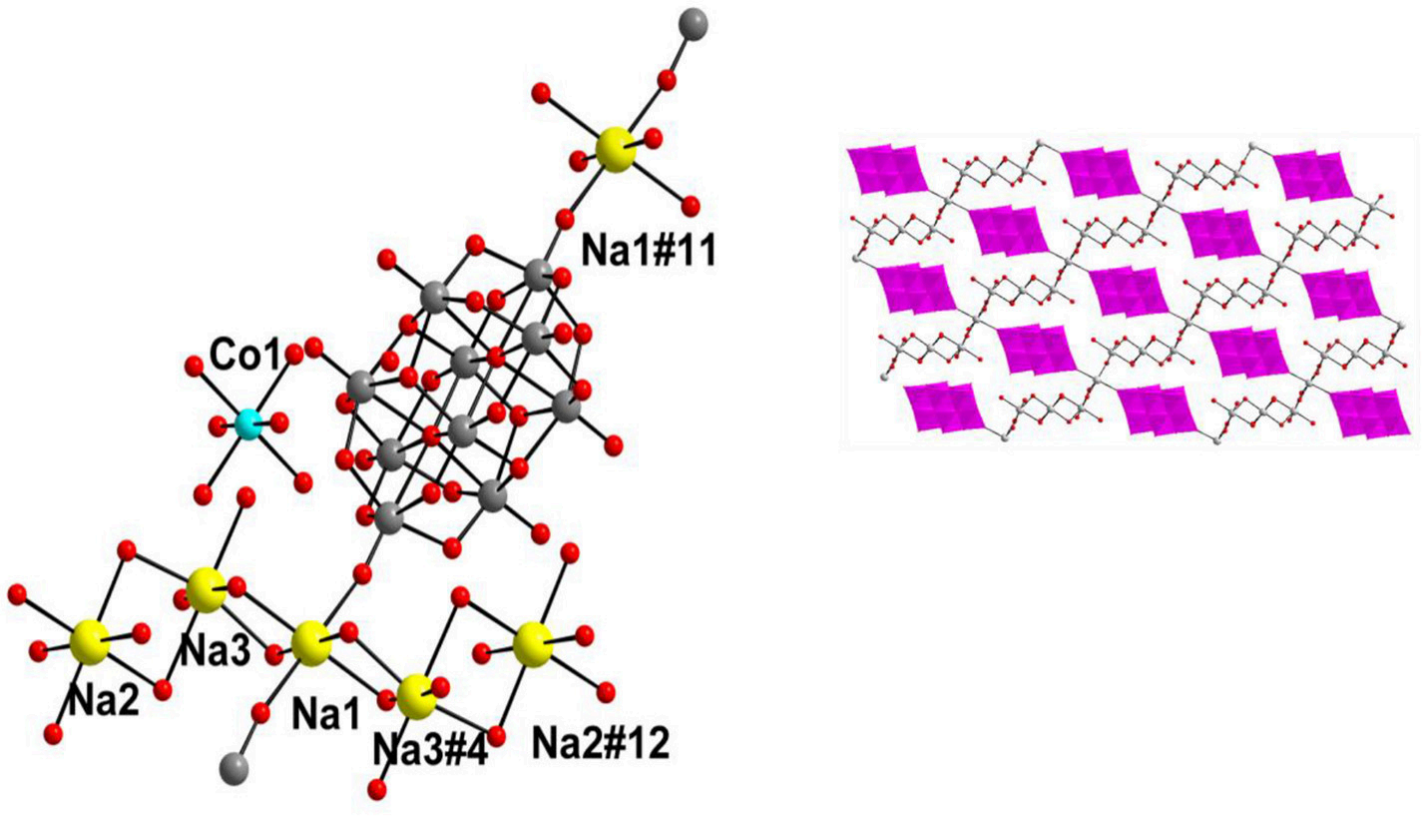
**Left:** Ball and stick representation of total molecule of the [Co(H_2_O)_6_][{Na_4_(H_2_O)_14_}{V_10_O_28_}]·4H_2_O (**1**) (Hydrogen atoms and solvent water molecules are omitted for clarity). color codes: Co, cyan; Na, yellow; O, red; V, light grey. **Right:** two-dimensional coordination polymer, formed in the crystal structure of compound **1**. Decavanadate clusters are shown in polyhedral representation (with pink color).

The synthesis and crystal structure of compound **1** has already been reported (Mestiri et al., 2013). Even then, we have described the crystal structure of compound **1** here in terms of supramolecular chemistry. The asymmetric unit of compound **1** reveals the presence of half of the decavanadate cluster, which supports the sodium-aqua-cluster complex cation {Na_2_(H_2_O)_7_}^2+^ by a coordinate covalent bond and an uncoordinated cobalt tri-aqua complex acting as the cation along with two lattice water molecules. The cationic species {Na_2_(H_2_O)_7_}^2+^ consists of three crystallographically independent sodium atoms, namely a Na3 (full occupancy), a Na2 (half occupancy) and a Na1 (half occupancy) in the asymmetric unit, which also includes the cobalt atom in the special position. Accordingly the full molecule is formulated as [Co(H_2_O)_6_][{Na_4_(H_2_O)_14_}{V_10_O_28_}]·4H_2_O (**1**). Thus, in compound **1**, the negative charges of {V_10_O_28_}^6−^ are counterbalanced by one coordination complex cation [Co(H_2_O)_6_]^2+^ and one alkali metal aqua cluster cation {Na_4_(H_2_O)_14_}^4+^. In the crystal structure, the sodium-aqua cluster cation {Na_4_(H_2_O)_14_}^4+^ is actually combination of {Na_3.5_(H_2_O)_12_}^3.5+^ and {Na(H_2_O)_2_}^0.5+^, whereby both cationic species are coordinated to the decavanadate cluster anion by coordinate covalent bonds. Five sodium atoms (two Na3, full occupancy + two Na2, half occupancy + one Na1, half occupancy) form the {Na_3.5_(H_2_O)_12_}^3.5+^ cluster cation, coordinating to the decavanadate anion through Na1 cation and the rest sodium atom Na1 (from {Na(H_2_O)_2_}^0.5+^ cationic species) coordinates to the same {V_10_} cluster from opposite side of the cluster anion. All sodium ions are octahedrally coordinated in the crystal structure. The coordination of {Na_4_(H_2_O)_14_}^4+^ cation to the {V_10_} cluster anion is shown in Figure 2 (left). We believe that, the pH 9 of the concerned synthesis mixture is important in forming such [Na_4_(H_2_O)_14_]^4+^ cluster.

**Figure 3:**
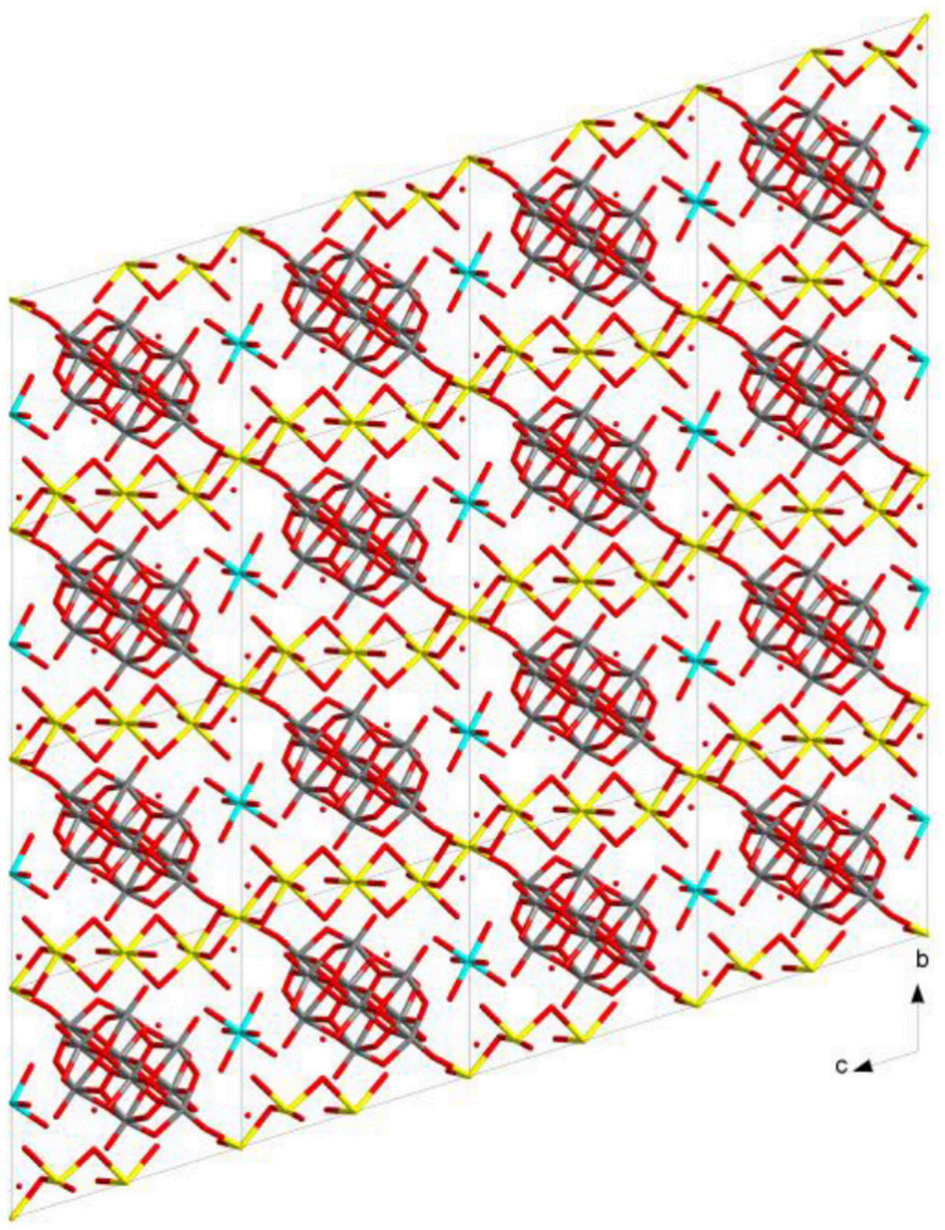
Molecular packing diagram in the crystal structure of compound **1**.

In the relevant crystal structure, this sodium aqua cluster cation self assembles to an infinite one-dimensional sodium water chain. These chains are laterally linked by {V_10_O_28_}^6−^ cluster anions through Na1 ion resulting in a two-dimensional pure inorganic coordination polymer as shown in Figure 2 (right). These two-dimensional sheets are stacked along crystallographic *a* axis as shown from the molecular packing diagram (Figure 3). The O–H···O hydrogen bonding situation in the crystal structure of [Co(H_2_O)_6_][{Na_4_(H_2_O)_14_}{V_10_O_28_}]·4H_2_O (**1**) has been described in Supplementary Figure 1 (Supporting Information). The relevant crystallographic data is available in Table S1 (Supporting Information). Tables S2, S3 (Supporting Information) have described bond distances along with bond angles and hydrogen bonding parameters respectively.

#### Compound [Zn(H_2_O)_6_][Na_3_(H_2_O)_14_][HV_10_O_28_]·4H_2_O (2)

**Figure 4:**
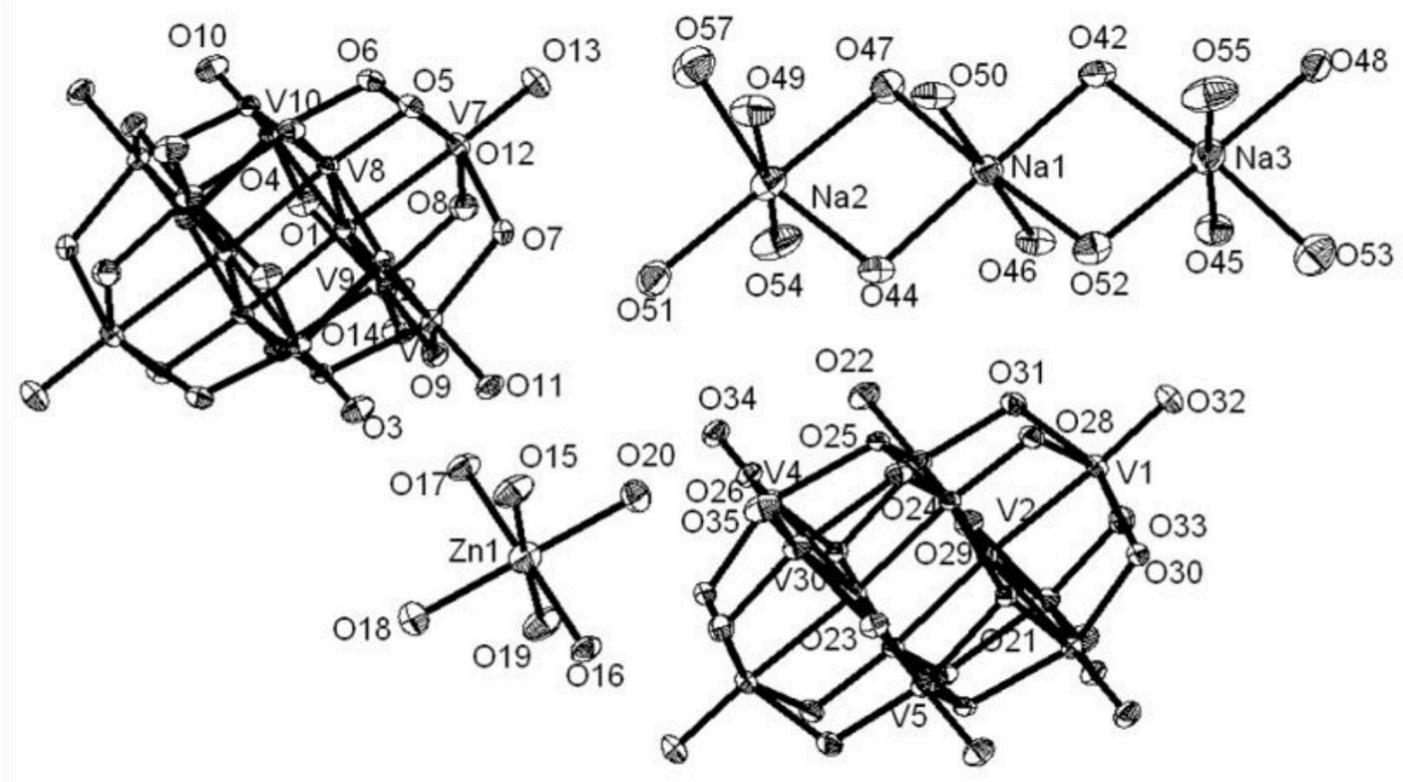
Thermal ellipsoidal diagram of asymmetric part of compound [Zn(H_2_O)_6_][Na_3_(H_2_O)_14_][HV_10_O_28_]·4H_2_O (**2**) with 30% probability (hydrogen atoms and solvent water molecules are omitted for clarity).

The asymmetric unit in the crystal structure of compound **2** consists of two one-half decavanadate clusters, one tri-sodium aqua-complex [Na_3_(H_2_O)_14_]^3+^, one zinc hexa-aqua complex [Zn(H_2_O)_6_]^2+^ and four solvent water molecules as shown in Figure 4. Thus the full molecule can be formulated as [Zn(H_2_O)_6_][Na_3_(H_2_O)_14_][HV_10_O_28_]·4H_2_O (**2**), in which decavanadate cluster is singly protonated, and the rest of the charge (−5) is counter-balanced by [Zn(H_2_O)_6_]^2+^ and [Na_3_(H_2_O)_14_]^3+^ cations. In the crystal structure, along with five lattice water molecules, fourteen water molecules are found to be coordinated with three sodium cations, resulting in the formation of [Na_3_(H_2_O)_14_]^3+^ cluster cation, in which the coordination of each sodium can be described by an octahedral arrangement of water molecules. The formation of this sodium-aqua cluster can be described by two terminal sodium ions (Na2 and Na3) and one middle sodium ion (Na1) as shown in Figure 4 (top right).

**Figure 5:**
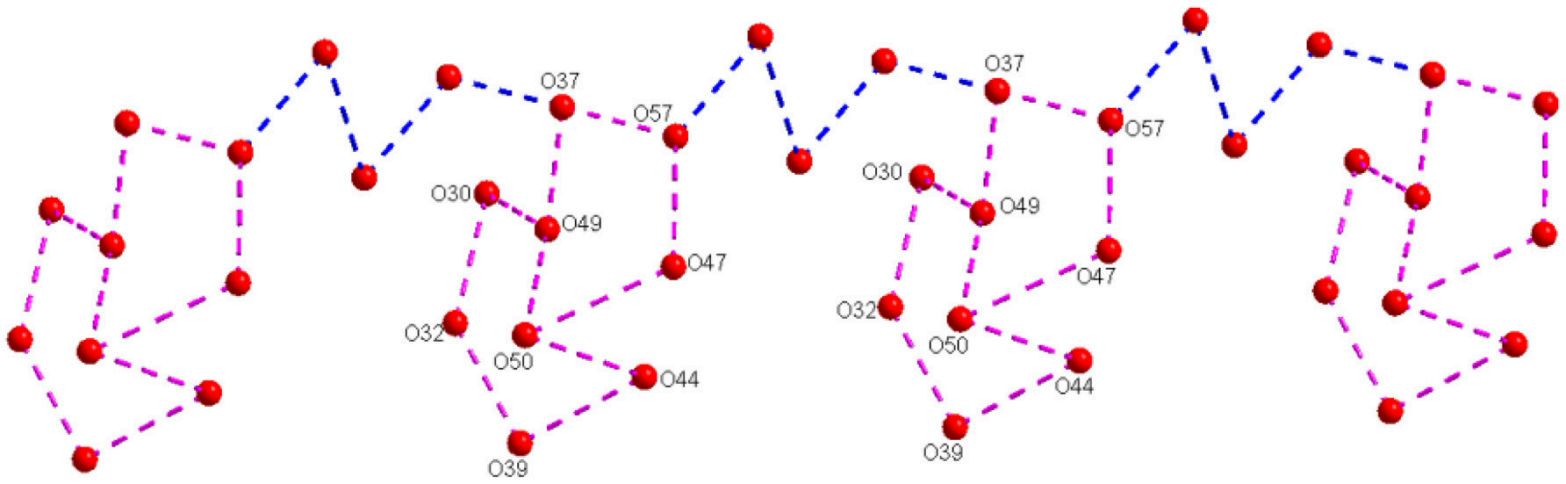
A chainlike water structure, built from water cluster due to O–H···O interactions among the lattice water and zinc and sodium coordinated water molecules in the crystal structure of compound [Zn(H_2_O)_6_][Na_3_(H_2_O)_14_][HV_10_O_28_]·4H_2_O (**2**).

**Figure 6:**
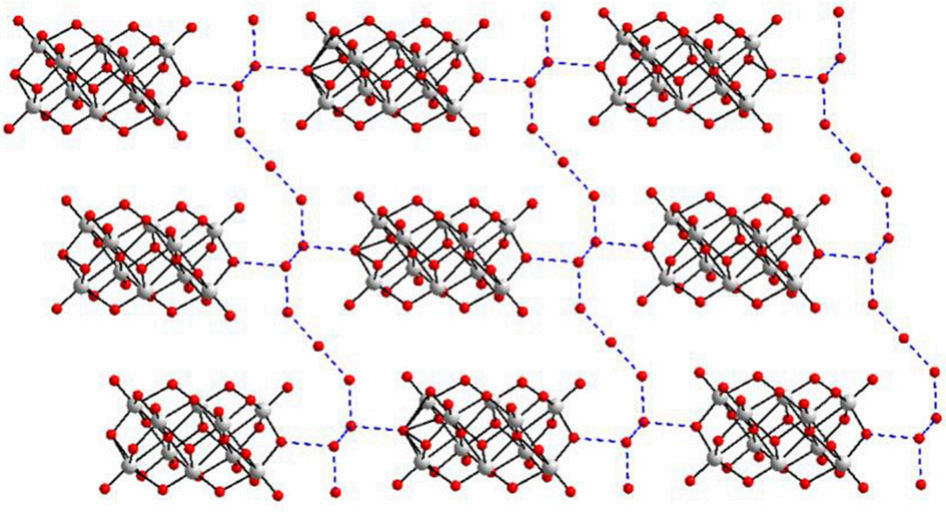
A hydrogen-bonded supramolecular array consisting of decavanadate cluster anion and water molecules including lattice waters and metal coordinated water molecules.

The middle sodium ion (Na1) is coordinated to four μ_2_-type bridging water molecules (O44, O47, O42, and O52) and two terminal water molecules (O46 and O50). Each of the terminal sodium ions is coordinated to two μ_2_-type bridging water molecules and four terminal water molecules as shown in Figure 4. Hydrogen atoms could not be located for all the water molecules in the concerned crystal. By taking O···O separation in the range of 2.779 Å to 3.211 Å, supramolecular chemistry of compound **2** is described. A supramolecular (H_2_O)_9_ cluster is found to be formed by zinc- and sodium-coordinated water molecules and solvent water molecules. These (H_2_O)_9_ clusters are further linked by (H_2_O)_3_ cluster (shown in blue dotted line) resulting in the formation of a chain-like arrangement as shown in Figure 5. The supramolecular O···O interactions between decavanadate cluster anions, the water chain and a (lattice) water dimer (O35···O35) lead to the generation of a supramolecular network as shown in Figure 6. This situation (Figure 6) can be depicted as inclusion of POV clusters in the water chain-network. In other words, it can be described that decavanadate cluster is stabilized in the pool of water.

#### [HMTAH]_2_[{Zn(H_2_O)_4_}_2_{V_10_O_28_}]·2H_2_O (3)

**Figure 7:**
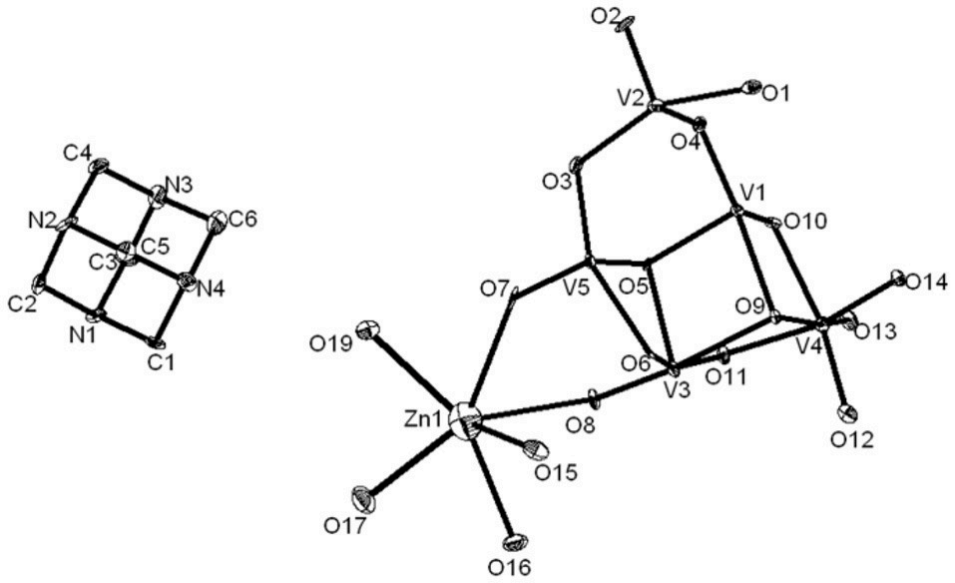
Thermal ellipsoidal diagram of asymmetric part of compound [HMTAH]_2_[{Zn(H_2_O)_4_}_2_{V_10_O_28_}]·2H_2_O (**3**).

**Figure 8:**
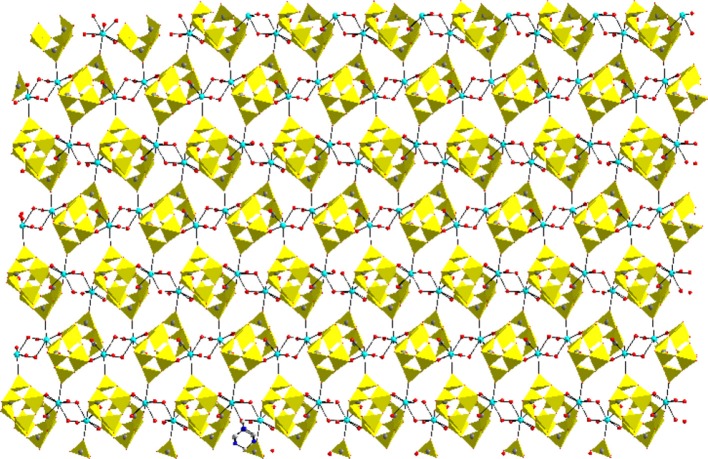
Two-dimensional coordination polymer, formed in the crystal structure of [HMTAH]_2_[{Zn(H_2_O)_4_}_2_{V_10_O_28_}]·2H_2_O (**3**). Color codes: V–O polyhedra, yellow; Zn, Cyan; O, red.

Compound **3**, that has been synthesized starting from sodium metavanadate, zinc nitrate and hexamine at pH 3.0 in a simple wet synthesis, crystallizes in monoclinic system with space group of *P2*_1_*/c*. The concerned asymmetric unit consists of half of the decavanadate anion, one HMTAH cation and one zinc aqua complex cation (Figure 7). Thus it has been formulated as [HMTAH]_2_[{Zn(H_2_O)_4_}_2_{V_10_O_28_}]·2H_2_O (**3**). In the crystal structure, decavanadate moiety is functionalized by a Zn(II)-aqua complex, in which Zn1 is coordinated to two terminal oxo groups (O7 and O8) of both sides of decavanadate anion as shown in Figure 7. Thus the coordination number of zinc in polyoxovanadate (POV) supported Zn(II)-tetra-aqua coordination complex is six (two terminal oxygen and four water molecules). Two such POV supported Zn(II) coordination complexes form a {Zn_2_} dimer through two {μ_2_-H_2_O} type water bridges. Inter-dimer-decavanadate cluster coordination results in the formation of a 2-dimensional coordination polymer as shown Figure 8. Hydrogen bonding environment around HMATAH^+^ moiety due to C–H···O interactions in the crystal structure of compound **3** is shown in Supplementary Figure 2. Hydrogen bond distances and angles are presented in Table S4.

#### Compound [{Co(3-amp)(H_2_O)_5_}_2_{3-ampH}_2_][V_10_O_28_]·6H_2_O (4)

**Figure 9:**
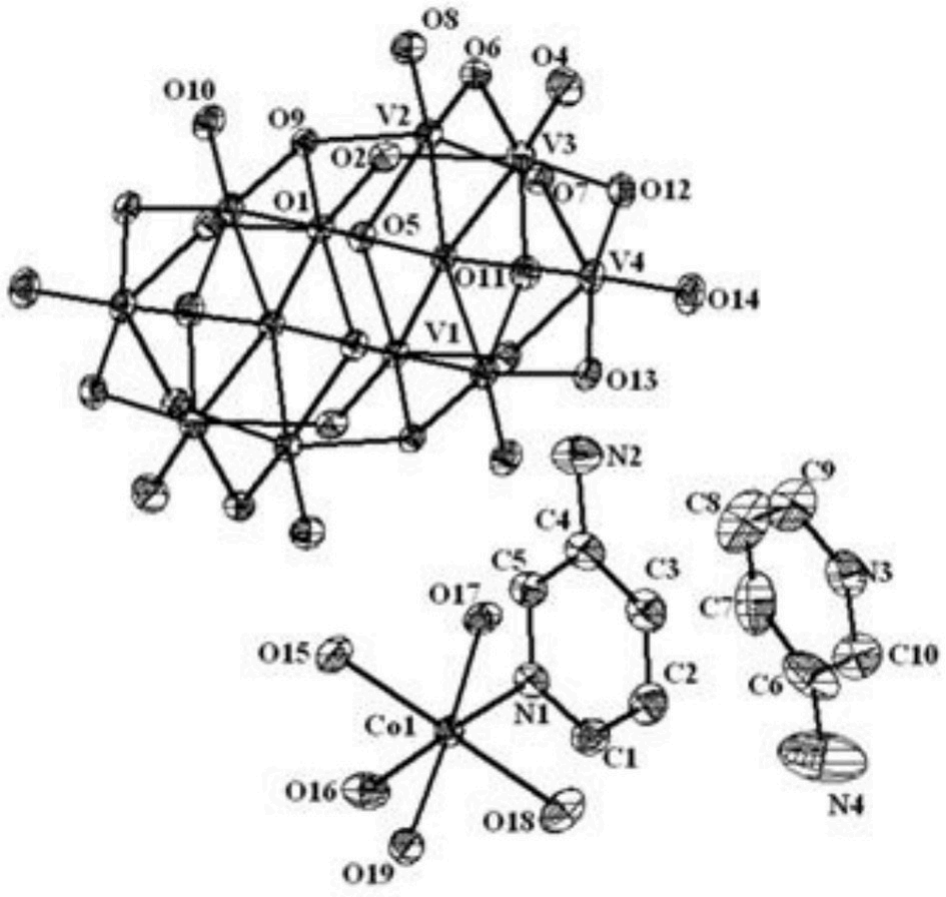
Thermal ellipsoidal diagram of [{Co(3-amp)(H_2_O)_5_}_2_{3-ampH}_2_][V_10_O_28_]·6H_2_O (**4**) with 30% probability (hydrogen atoms are omitted for clarity).

**Figure 10:**
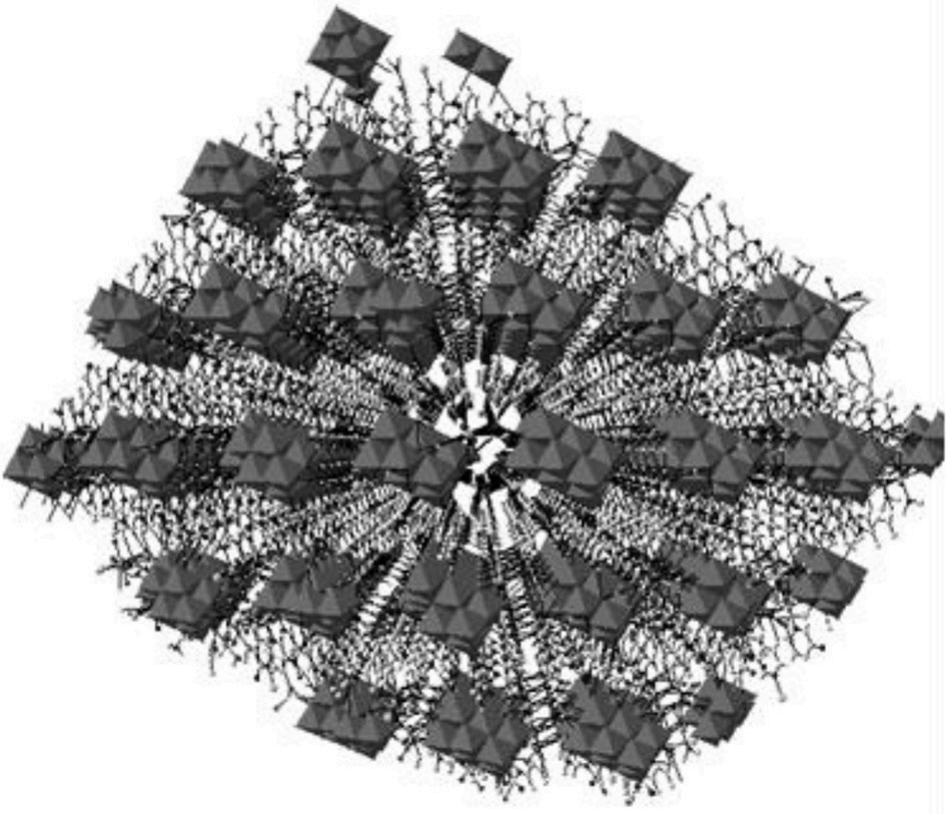
A 3-dimensional framework (a perspective view), generated due to C–H···O hydrogen bonding interactions between cation and decavanadate anion in the crystal of compound [{Co(3-amp)(H_2_O)_5_}_2_{3-ampH}_2_][V_10_O_28_]·6H_2_O (**4**).

A discrete inorganic-organic hybrid material [Co(3-amp)(H_2_O)_5_]_2_[3-ampH]_2_{V_10_O_28_}·6H_2_O (**4**) containing a cobalt complex, a decavanadate cluster anion, aminopyridinium cation and lattice water molecules, has been isolated with 3-aminopyridine in an aqueous medium in an one pot synthesis. Crystal system is confined with triclinic *P*-1 space group. The relevant asymmetric unit, as shown in Figure 9, reveals the presence of half of the decavanadate anionic cluster, one molecule of protonated 3-aminopyridine [3-ampH]^+^ and a cobalt coordination complex {Co(3-amp)(H_2_O)_5_}^2+^. Apart from columbic interaction between cation and anionic species, non-covalent interactions are also responsible for stability of the compound **4**. In the crystal structure, a three-dimensional framework has been built due to C–H···O hydrogen bonding interactions between the cationic part and decavanadate anionic cluster as shown in Figure 10. Hydrogen bonding situation around the 3-aminopryridines (coordinated as well as cation) and water molecule, is shown in Supplementary Figure 3 and their hydrogen bonding distances and angles are shown in Table S5 including pertinent symmetry operations. We found weak π-π interactions among the molecules of 3-aminopyridine (see Supplementary Figure 4).

#### Compound [4-ampH]_10_[{Na(H_2_O)_6_} {HV_10_O_28_}] [V_10_O_28_] ·15H_2_O (5)

**Figure 11:**
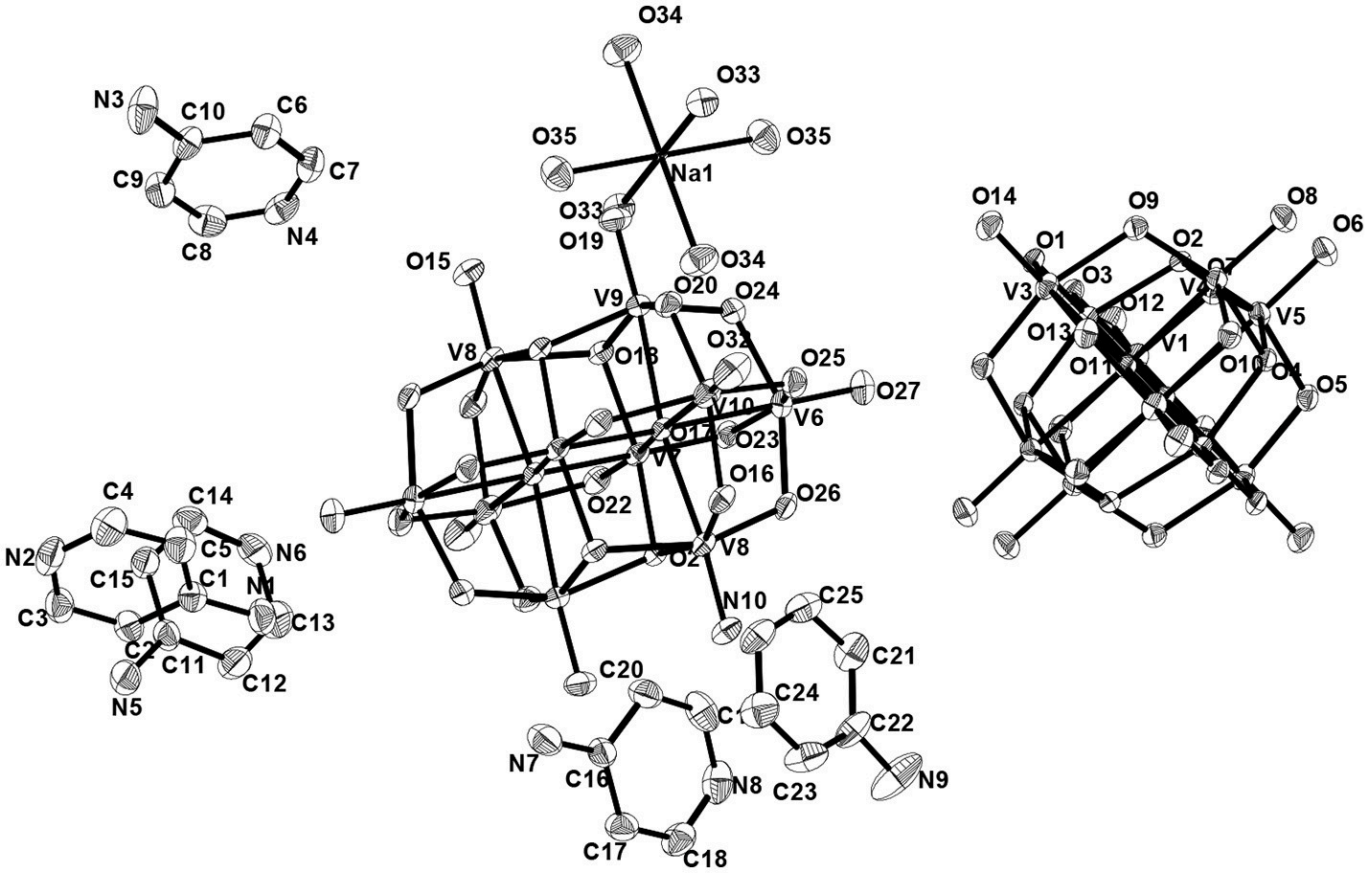
Thermal ellipsoidal Thermal Ellipsoidal diagram of [4-ampH]_10_[{Na(H_2_O)_6_} {HV_10_O_28_}] [V_10_O_28_] ·15H_2_O (**5**) with 30% probability (hydrogen atoms are omitted for clarity).

**Figure 12:**
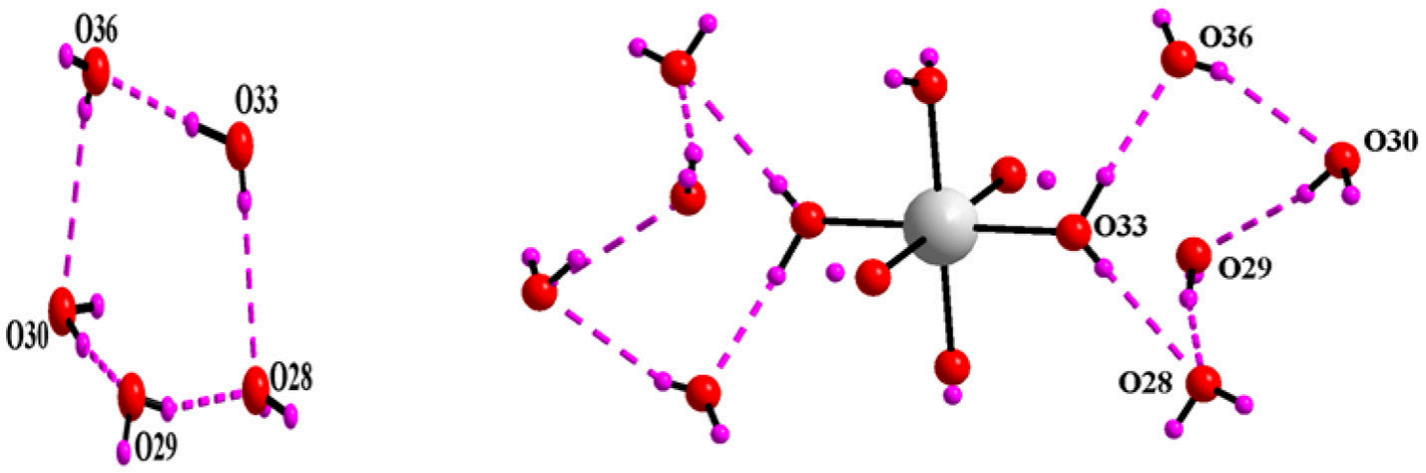
**Left:** cyclic water pentamer is generated due to O–H···O interactions in [4-ampH]_10_[{Na(H_2_O)_6_} {HV_10_O_28_}] [V_10_O_28_] ·15H_2_O (**5**); **right:** dumbbell shape diagram due to O–H···O interaction in 6 involving {Na(H_2_O)_6_} coordination complex. Color codes: O, red; Na, grey; H, purple.

Asymmetric unit of the crystal structure of compound **5** reveals that two independent halves of decavanadate anionic cluster [V_10_O_28_]^6−^ are assembled with the five cation molecules of 4-ampH^+^ and a Na(H_2_O)_3_ moiety with sodium in special position. ORTEP diagram of the [4-ampH]_10_[{Na(H_2_O)_6_} {HV_10_O_28_}] [V_10_O_28_] ·15H_2_O (**5**) is shown in the Figure 11. We have found ten lattice water molecules in the crystal structure of **5** and a cyclic water pentamer is generated due of the O–H···O hydrogen bonding interactions among the water molecules: O28, O29, O30, O33, and O36 as shown in Figure 12, left. Two such water pentamers are connected with a central sodium hexa-aqua complex *via* Na–O–H···O (pentamer) interactions resulting in the generation of a dumbbell-shaped-supramolecular architecture as presented in Figure 12, right. The relevant crystallographic data is provided in Table 2. And C–H···O and O–H···O hydrogen bonding interactions around {N1N2}, {N3N4}, {N5N6}, {N7N8}, {N9N10}, and water moieties in the crystal structure of **5** are shown in Supplementary Figure 5 and pertinent hydrogen bond distances and angles are listed in the Table S6 including symmetry operations.

#### Compound [{4-ampH}_6_{Co(H_2_O)_6_}_3_][V_10_O_28_]_2_·14H_2_O (6)

**Figure 13:**
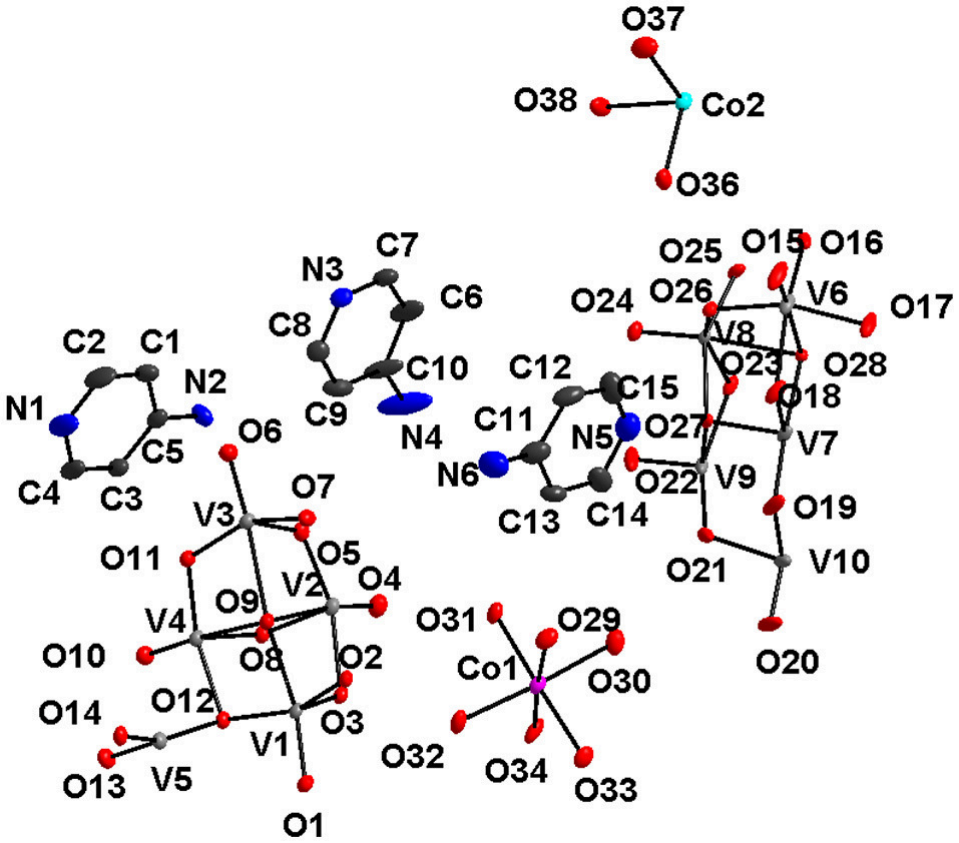
Thermal ellipsoidal plot of the asymmetric unit of compound [{4-ampH}_6_{Co(H2O)_6_}_3_][V10O28]_2_·14H_2_O (6) (hydrogen atoms and solvent water molecules are omitted for clarity). Color codes: O, red; V, medium grey; C, dark grey; Co, cyan.

**Figure 14:**
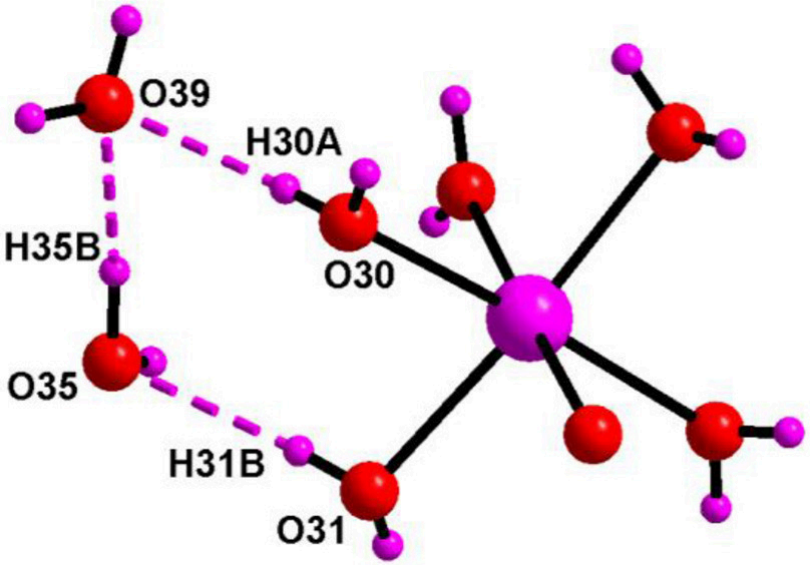
Supramolecular water tetramer, generated due to O–H···O interactions in the crystal of [{4-ampH}_6_{Co(H_2_O)_6_}_3_][V_10_O_28_]_2_·14H_2_O (**6**) involving the cobalt complex. Color codes: O, red; H and Co, purple.

The asymmetric unit of compound **6** (Figure 13) consists of two independent haves of decavanadate {V_10_O_28_}^6−^ cluster anion, 1.5 molecules of [Co(H_2_O)_6_]^2+^, three protonated 4-aminopyridine and seven solvent water molecules. Accordingly, this is formulated as [{4-ampH}_6_{Co(H_2_O)_6_}_3_][V_10_O_28_]_2_·14H_2_O (**6**). In the crystal structure, coordinated water molecules and lattice water molecules are non-covalently interacted through hydrogen bonding, generating a water tetramer (O30, O39, O35, O31) with O···H distances of 2.01 Å, 1.842 Å, and 2.077 Å, respectively (Figure 14) and each end oxygen atoms of the water tetramer, i.e., O30 and O31, is linked to cobalt center of the cobalt-hexa-aqua complex. Hydrogen bonding situation around {N1N2}, {N3N4}, {N5N6}, {N7N8}, {N9N10}, {Co} and water in the crystal structure of **6** is shown in Supplementary Figure 6, which is explained based on C–H···O, O–H···O, and N–H···O interactions. The relevant hydrogen bond distances and angles are listed in the Table S7 in the section of Supplementary Material.

#### Compound [{4-ampH}_10_{Zn(H_2_O)_6_}][V_10_O_28_]_2_ ·10H_2_O (7)

**Figure 15:**
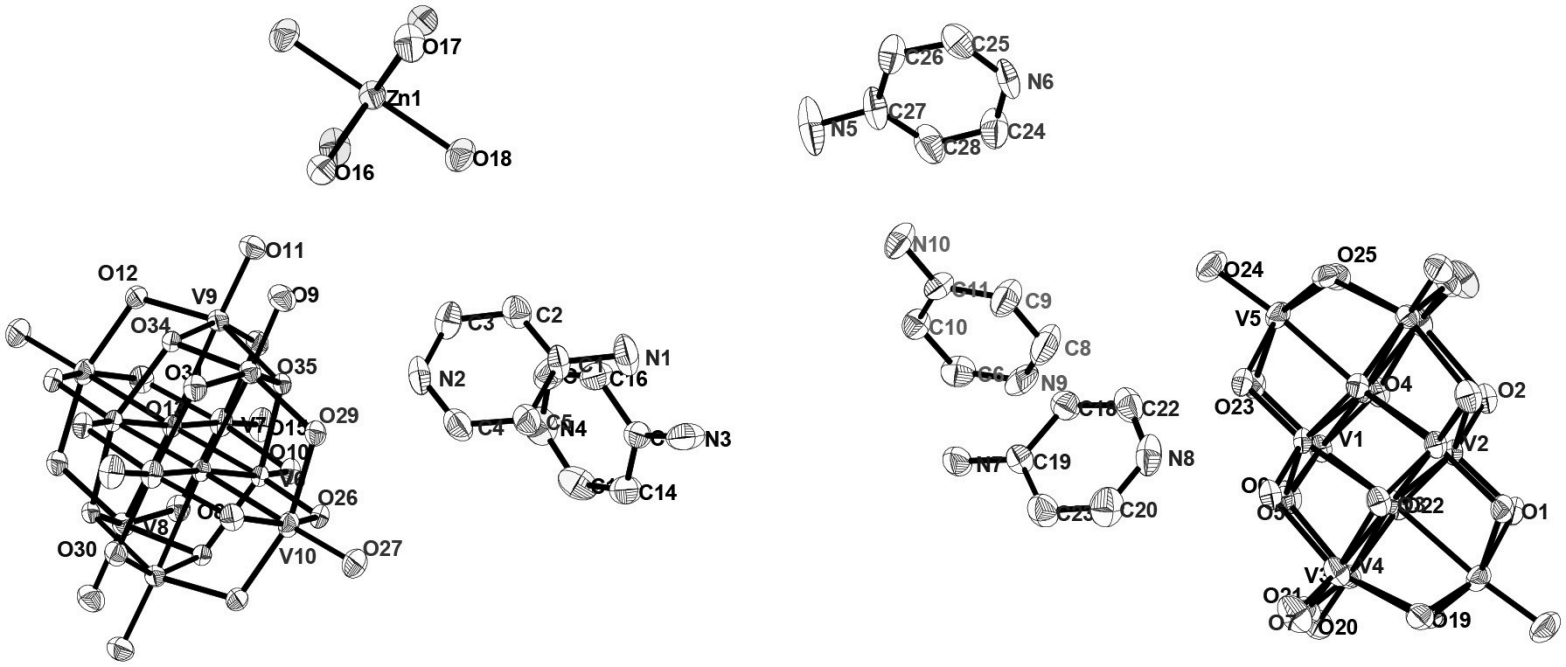
ORTEP diagram in the crystal structure of compound [{4-ampH}_10_{Zn(H_2_O)_6_}][V_10_O_28_]_2_ ·10H_2_O (**7**) with 30% probability (hydrogen atoms and solvent water molecules are omitted for clarity).

**Figure 16:**
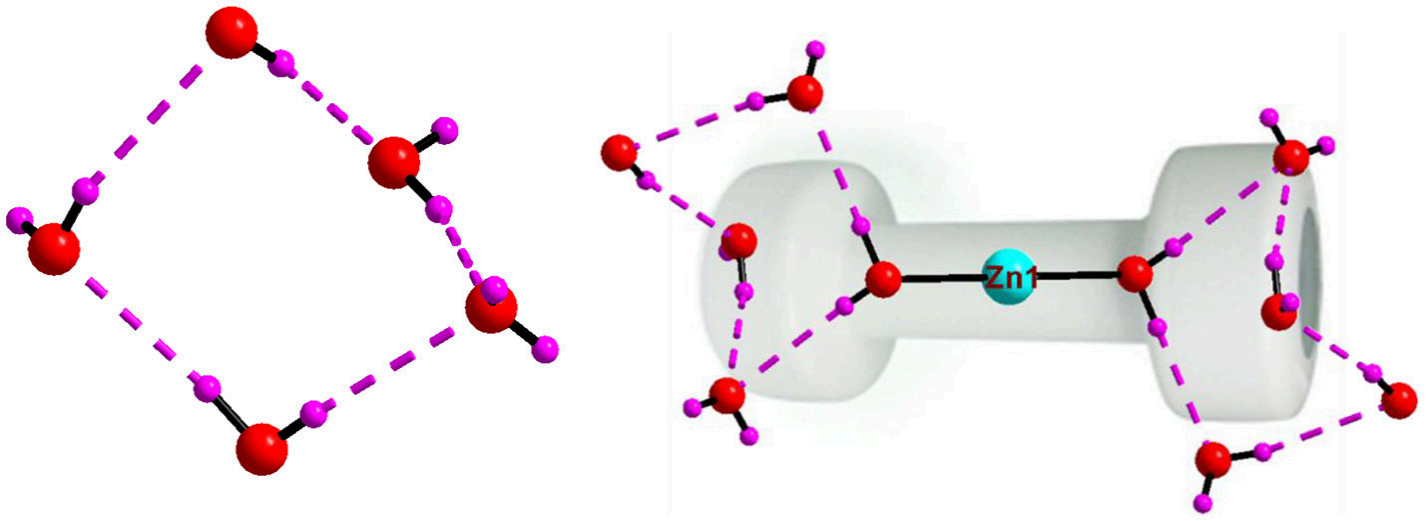
**Left:** cyclic water pentamer, generated due to O–H···O interactions in compound [{4-ampH}_10_{Zn(H_2_O)_6_}][V_10_O_28_]_2_ ·10H_2_O (**7**); **right:** dumbbell shape diagram due to O–H···O interactions in **7** involving {Zn(H_2_O)_6_} coordination complex and water pentamers. Color codes: O, red; Zn, cyan; H, purple.

The crystal structure of [{4-ampH}_10_{Zn(H_2_O)_6_}][V_10_O_28_]_2_ ·10H_2_O (**7**) has been characterized with the monoclinic, *P2*_1_*/c* space group. It consists of two clusters of decavanadate anion {V_10_O_28_}^6−^, ten molecules of protonated 4-aminopyridine and one unit of zinc(II)-hexa-aqua complex [Zn(H_2_O)_6_]^2+^. In addition, ten lattice water molecules are crystallized in the crystal structure of compound **7**. Thermal ellipsoidal diagram of compound **7** is presented in Figure 15. In the crystal structure, lattice water molecules (O36, O37, O40, O38), and coordinated water molecule (O16) are non-covalently interacted due to O–H···O hydrogen bonding interactions resulting in the generation of a cyclic water pentamer, that has been represented in Figure 16, left. In this supramolecular water pentamer, since the O16 water is the Zn(II)-coordinated water molecule and it is equivalent to another O16 by a symmetry operation coordinated to same Zn(II), it is possible to have two water pentamers connected by a central Zn(II) ion resulting in dumbbell-like construction as shown in Figure 16, right (O···H distances lie between 2.007 Å and 2.158 Å). Hydrogen bonding situations around {N1N2}, {N3N4}, {N5N6}, {N7N8}, {N9N10}, {Zn} and water due to C–H···O, N–H···O and O–H···O interactions in the crystal structure of **7** are shown in Supplementary Figure 7 and relevant hydrogen bond distances and angles are given in Table S8 with symmetry codes.

### Understanding decavanadate-based mineralogy

Until now, more than 12 decavanadate based minerals have been known (Table 3) and interestingly, many of them have been characterized by crystallography. Out of seven described compounds in this work, two compounds [Co(H_2_O)_6_][{Na_4_(H_2_O)_14_}{V_10_O_28_}]·4H_2_O (**1**) and [Zn(H_2_O)_6_] [Na_3_(H_2_O)_14_] [HV_10_O_28_]·4H_2_O (**2**) have direct relevance to the decavanadate-based mineral. For example, the mineral kokinosite, Na_2_Ca_2_(V_10_O_28_)•24H_2_O (Kampf et al., 2014) and compound [Co(H_2_O)_6_][{Na_4_(H_2_O)_14_}{V_10_O_28_}]·4H_2_O (**1**) (the formula of which can also be written as Na_4_Co (V_10_O_28_)•24H_2_O) can be discussed in the context of the microenvironment of the isopolyanion [V_10_O_28_]^6−^ in this synthesized compound **1** and in kokinosite. Both compound **1** and the kokinosite mineral have similar / comparable crystal cell parameters and crystallize in the same space group. In the crystal structure of compound **1**, we have found the abundance of {Na_4_(H_2_O)_14_}^4+^ liner-shaped water-bridged cluster (per formula unit), which is coordinated to the decavanadate cluster anion through terminal oxygen of the polyanion. In other words, in the synthesized compound **1**, decavanadate supported sodium-water cluster {Na_4_(H_2_O)_14_}^4+^ exists (Figure 2). In the relevant crystal structure, the interlinking of these clusters results in the infinite sodium-water chain. These sodium-water chains are laterally linked by the decavanadate anions to form a two-dimensional coordination polymeric structure; [Co(H_2_O)_6_]^2+^ remains as an isolated discrete coordination complex cation located in the void space (Figure 3) to counterbalance the negative charges of the decavanadate anion. In the language of decavanadate mineral crystallography, these cations (e.g., sodium and cobalt ions in compound **1**) are called interstitial units (Hughes et al., 2002, 2005, 2008; Colombo et al., 2011; Kampf et al., 2011b, 2013b, 2014). For example, in the crystal structure of the kokinosite mineral (Kampf et al., 2014), the interstitial unit has a composition of (Na_2_Ca_2_•24H_2_O)^6+^ and the decavanadate anion (V_10_O_28_)^6−^ forms the structural unit of the mineral. In the relevant crystal structure (kokinosite), the decavandate cluster anions (structural units) are linked by Na(H_2_O)_6_ octahedra (interstitial units) and Ca(H_2_O)_8_ polyhedra (interstitial units) that themselves link into infinite chains by edge and corner sharing. So in this case, Ca(H_2_O)_8_ is part of the infinite chain (unlike Co(H_2_O)_6_ in compound **1**, that remains as a isolated complex cation). Overall, both structures are comparable.

Compound [Zn(H_2_O)_6_][Na_3_(H_2_O)_14_][HV_10_O_28_]·4H_2_O (**2**), synthesized and characterized in this work, is formulated with a mono-protonated decavanadate cluster anion [HV_10_O_28_]^5−^. In 2011, Kampf et al. reported diprotonated decavanadate based mineral, gunterite Na_4_(H_2_V_10_O_28_) •22H_2_O (Kampf et al., 2011b). In both the crystal structures, the proton on the decavanadate anion could not be located, but formulated from elemental analyses and crystallographic investigation on the cationic part. In the crystal structure of compound **2**, a sodium trimer-water cluster [Na_3_(H_2_O)_14_]^3+^ per formula unit is characterized, which unlike compound 1 is not coordinated to decavanadate anion. But in the case of the gunterite mineral, the sodium water cluster is coordinated to the polyanion (Kampf et al., 2011b). In the crystal structure of synthesized compound **2**, the [Zn(H_2_O)_6_]^2+^ moiety remains as an isolated discrete coordination complex cation, counter balancing the residual charge of the decavanadate anion. In most of these mineral structures, solvent waters are found disordered; on the other hand, in most of the synthesized compounds (present work), lattice waters are characterized as forming supramolecular water clusters. So far, no decavanadate based minerals are found, in which the interstitial cation is a transition metal-aqua complex cation, as synthesized in the present work. The present synthesis work predicts that the formation of such transition metal cation associated decavanadate minerals is possible and we expect that transition metal decavanadate minerals will be discovered in future that may feature the presence of discrete hexa-aqua transition metal coordination complex as an interstitial cation, as found in the present model study.

## Conclusions

We have described the synthesis and characterization of seven decavanadate containing compounds [Co(H_2_O)_6_] [{Na_4_(H_2_O)_14_} {V_10_O_28_}] ·4H_2_O (**1**), [Zn(H_2_O)_6_] [Na_3_(H_2_O)_14_] [HV_10_O_28_] ·4H_2_O (**2**), [HMTAH]_2_[{Zn(H_2_O) _4_}_2_{V_10_O_28_}] ·2H_2_O (**3**), [{Co(3-amp)(H_2_O)_5_} _2_{3-ampH}_2_] [V_10_O_28_] ·6H_2_O (**4**), [4-ampH]_10_[{Na(H_2_O)_6_} {HV_10_O_28_}] [V_10_O_28_] ·15H_2_O (**5**), [{4-ampH}_6_ {Co(H_2_O)_6_}_3_] [V_10_O_28_]_2_·14H_2_O (**6**), [{4-ampH}_10_ {Zn(H_2_O)_6_}] [V_10_O_28_]_2_ ·10H_2_O (**7**). In this report, we have described detailed supramolecular chemistry of **1**−7. In some of their crystal structures, non-covalent interactions among the lattice water molecules and metal coordinated water molecules lead to the formation of supramolecular water clusters. Thus the microenvironment of the decavanadate cluster anion in diverse cation matrices includes the formation of ordered water structures. We have also observed that, in these decavanadate based systems, the transition metal aqua complexes like to remain as discrete coordination complex cation, whereas the alkali metal aqua complexes like to aggregate to dimer, trimer, tetramer, infinite chain etc. These alkali metal water aggregations mimic the interstitial units of natural minerals of decavanadates. We have discussed the supramolecular chemistry of the synthesized systems comparing the microenvironment of decavanadate cluster anion, found in natural minerals.

## Author contributions

SA was Ph.D. student and SD is his Ph.D. supervisor. SA synthesized and characterized the compounds, described in this manuscript under the supervision of SD. Both SA and SD analyzed the data together. This manuscript has been written by SD mainly, but it was shown to SA. SA has agreed with the content of the manuscript.

### Conflict of interest statement

The authors declare that the research was conducted in the absence of any commercial or financial relationships that could be construed as a potential conflict of interest.
