# Supplementary material for: A Versatile Polyoxovanadate in Diverse Cation Matrices: A Supramolecular Perspective
Source: Front Chem. 2018 Oct 16;6:469. doi: 10.3389/fchem.2018.00469 (PMC6198037; doi:10.3389/fchem.2018.00469)
Supplement: Supplementary file 1 [file Data_Sheet_1.pdf]

Supplementary Material

A Versatile Polyoxovanadate in Diverse Cation Matrices: A Supramolecular Perspective

*A. Srinivasa Rao and Samar K Das**

* **Correspondence:** Corresponding Author: skdas@uohyd.ac.in

1 Supplementary Figures and Tables

1.1 Supplementary Figures

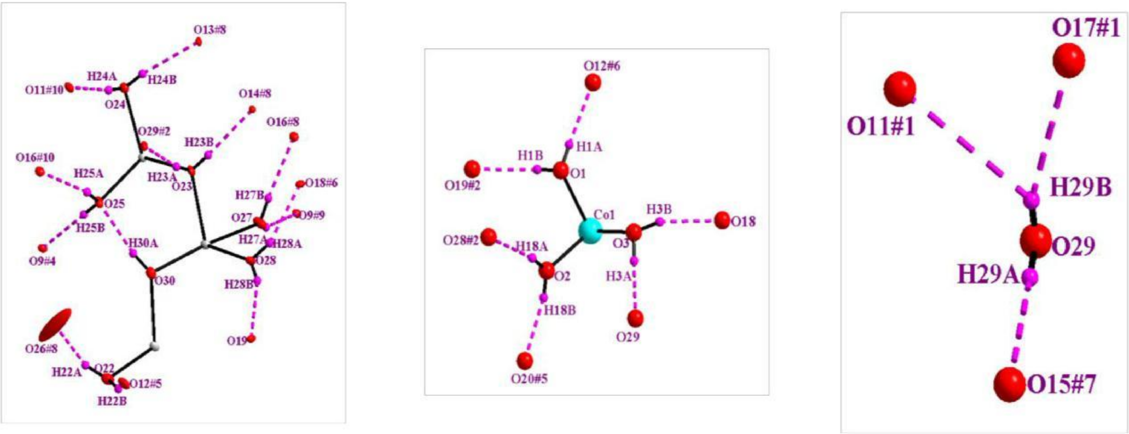

SF1a **SF1b** **SF1c**

Supplementary Figure 1. Hydrogen bonding situation around {Na}, {Co} and water motifs in compound $[Co(H_2O)_6][\{Na_4(H_2O)_{14}\}\{V_{10}O_{28}\}]\cdot 4H_2O$ (**1**). Color codes: Na, grey, Co, green; O, red, H, purple. symmetry codes: Symmetry codes: #1, -x+2, -y+1, -z+1; #2, -x+1, -y+1, -z; #3, -x+1, -y, - z-1, #4 -x+1, -y, -z; #5 x-1, y, z; #6 -x+2, -y+1, -z; #7 -x+1, -y+1, -z+1, #8 x, y, z-1; #9, -x+2, -y, - z; #10 x-1, y, z-1.

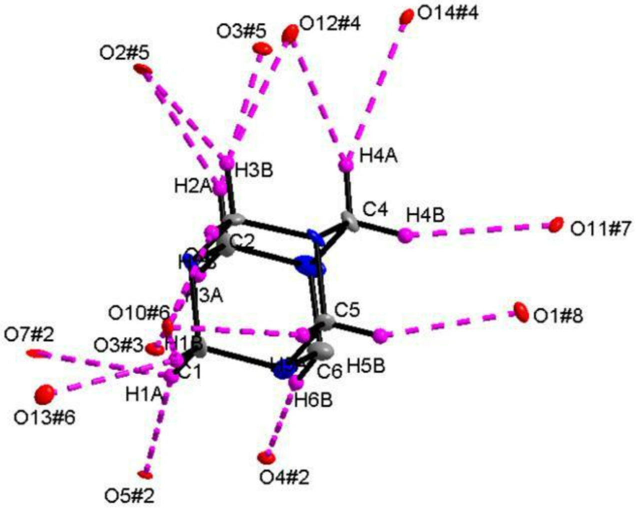

Supplementary Figure 2. Hydrogen bonding environment around the organic cation $[HMATAH]^{1+}$ in $[HMTAH]_2[\{Zn(H_2O)_4\}_2\{V_{10}O_{28}\}]\cdot 2H_2O$ (**3**). Symmetry codes: #1, -x+1,-y+1,-z; #2, x,-y+3/2,z+1/2; #3, x,-y+3/2,z-1/2; #4, x+1,-y+3/2,z+3/2; #5, -x+2,y+1/2,-z+3/2; #6, x, y, z+1, #7 -x+1, y, z+1; #8, x+2,-y+1,-z+1. Color codes: O, red; C, grey; H, purple; N, blue.

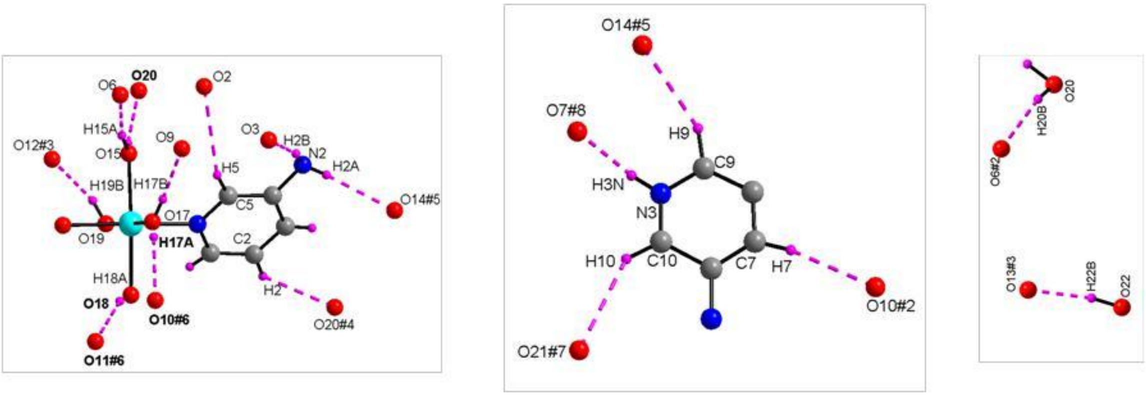

Supplementary Figure 3. Hydrogen bonding environment around 3-aminopyridines and water moieties in compound $[\{Co(3\text{-amp})(H_2O)_5\}_2\{3\text{-ampH}\}_2][V_{10}O_{28}]\cdot 6H_2O$ (**4**). Symmetry codes. #1, - x+1,-y+2,-z+2; #2, -x+1,-y+1,-z+2; #3, x,y-1,z; #4, -x+1,-y+1,-z+1; #5, -x+1,-y+2,-z+1; #6, - x+2,-y+2,-z+2; #7, -x+2,-y+1,-z+1; #8, x,y,z-1. Color codes: O, red; C, grey; H, purple; N, blue.

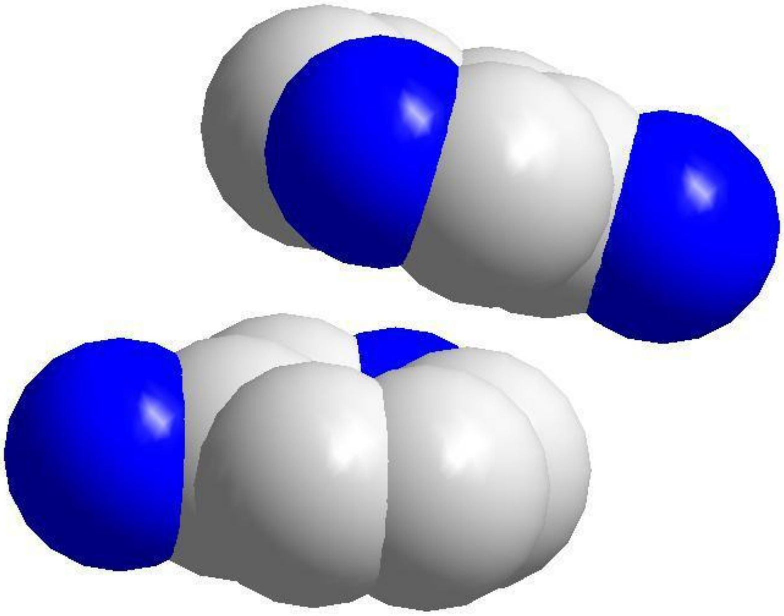

Supplementary Figure 4. Arrangement (space filling presentation) between two 3-aminopyridine molecules due to π–π interactions (3.718 Å) in the crystal structure of compound $[\{Co(3amp)(H_2O)_5\}_2\{3\text{-}ampH\}_2][V_{10}O_{28}]\cdot 6H_2O$ (**4**) (hydrogen atoms are omitted for clarity). Color codes: C, medium grey; N, blue.

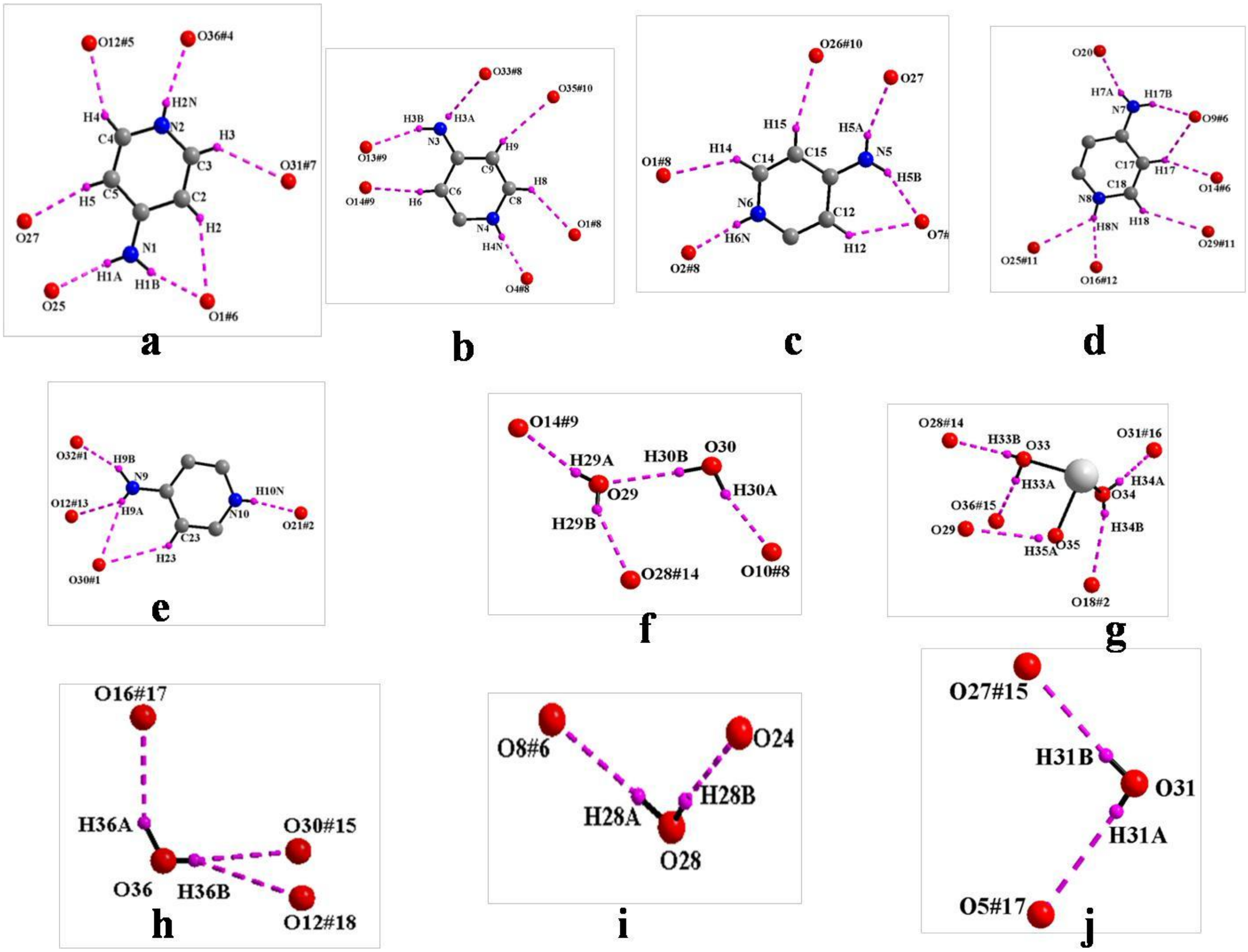

Supplementary Figure 5. Hydrogen bonding environment around (a) {N1N2}, (b) {N3N4}, (c) {N5N6}, (d) {N7N8}, (e) {N9N10} and (f-j) water moieties in the crystal structure of compound [4-ampH]$_{10}$[{Na(H$_2$O)$_6$} {HV$_{10}$O$_{28}$}] [V$_{10}$O$_{28}$] ·15H$_2$O (**5**). Symmetry codes: #1, -x,-y,-z; #2, -x+1,-y+1,-z; #3, -x,-y+1,-z; #4,x,-y+3/2,z-1/2; #5, x+1,y+1,z; #6, -x+1,y+1/2,-z+1/2; #7, -x+1,y+1/2,-z+3/2; #8, -x,y+1/2,-z+1/2; #9, x,y+1,z; #10, x,-y+3/2,z+1/2; #11, -x+1,y-1/2,-z+1/2; #12, x,-y+1/2,z+1/2; #13, x+1,y,z; #14, x-1,y,z; #15, -x+1,-y+1,-z+1; #16, x,y,z-1; #17, x,y,z+1; #18, x+1,-y+1/2,z+1/2; Color codes: O, red; C, grey; H, purple, N, blue.

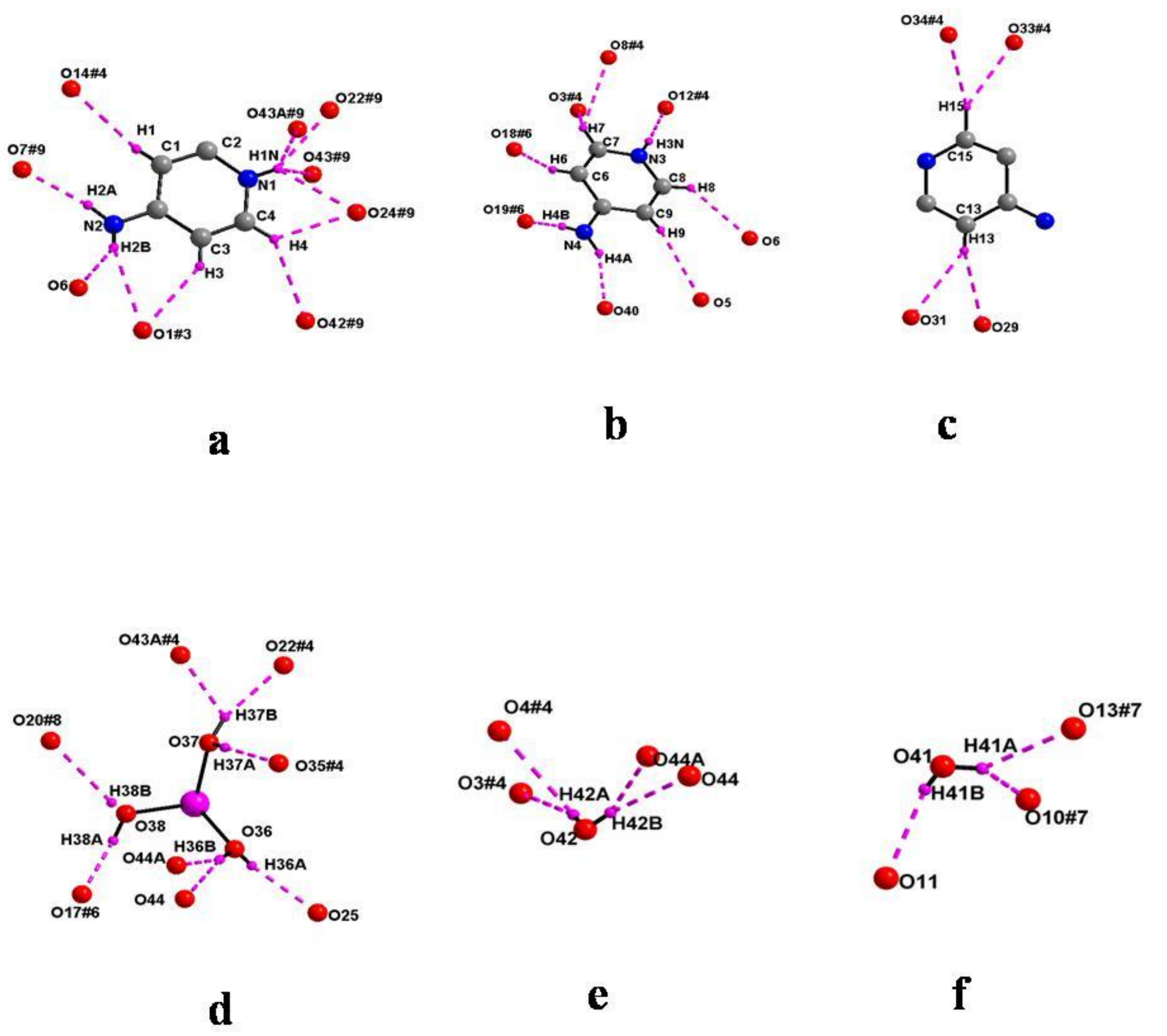

Supplementary Figure 6. Hydrogen bonding environment around (a) {N1N2}, (b) {N3N4}, (c) {N5N6}, (d) {Co} and (e-f) water moieties in the crystal structure of compound [{4-ampH}$_6${Co(H_2O)$_6$}$_3$][$V_{10}O_{28}$]$_2$·10H_2O (**6**). Symmetry codes: #1, -x,-y,-z; #2, -x+1,-y+1,-z; #3, -x,-y+1,-z; #4,x,-y+3/2,z-1/2; #5, x+1,y+1,z; #6, -x+1,y+1/2,-z+1/2; #7, -x+1,y+1/2,-z+3/2; #8, -x,y+1/2,-z+1/2; #9, x,y+1,z; #10, x,-y+3/2,z+1/2; #11, -x+1,y-1/2,-z+1/2; #12, x,-y+1/2,z+1/2; #13, x+1,y,z; #14, x-1,y,z; #15, -x+1,-y+1,-z+1; #16, x,y,z-1; #17, x,y,z+1; #18, x+1,-y+1/2,z+1/2; Color codes: O, red; C, grey; H, purple, N, blue.

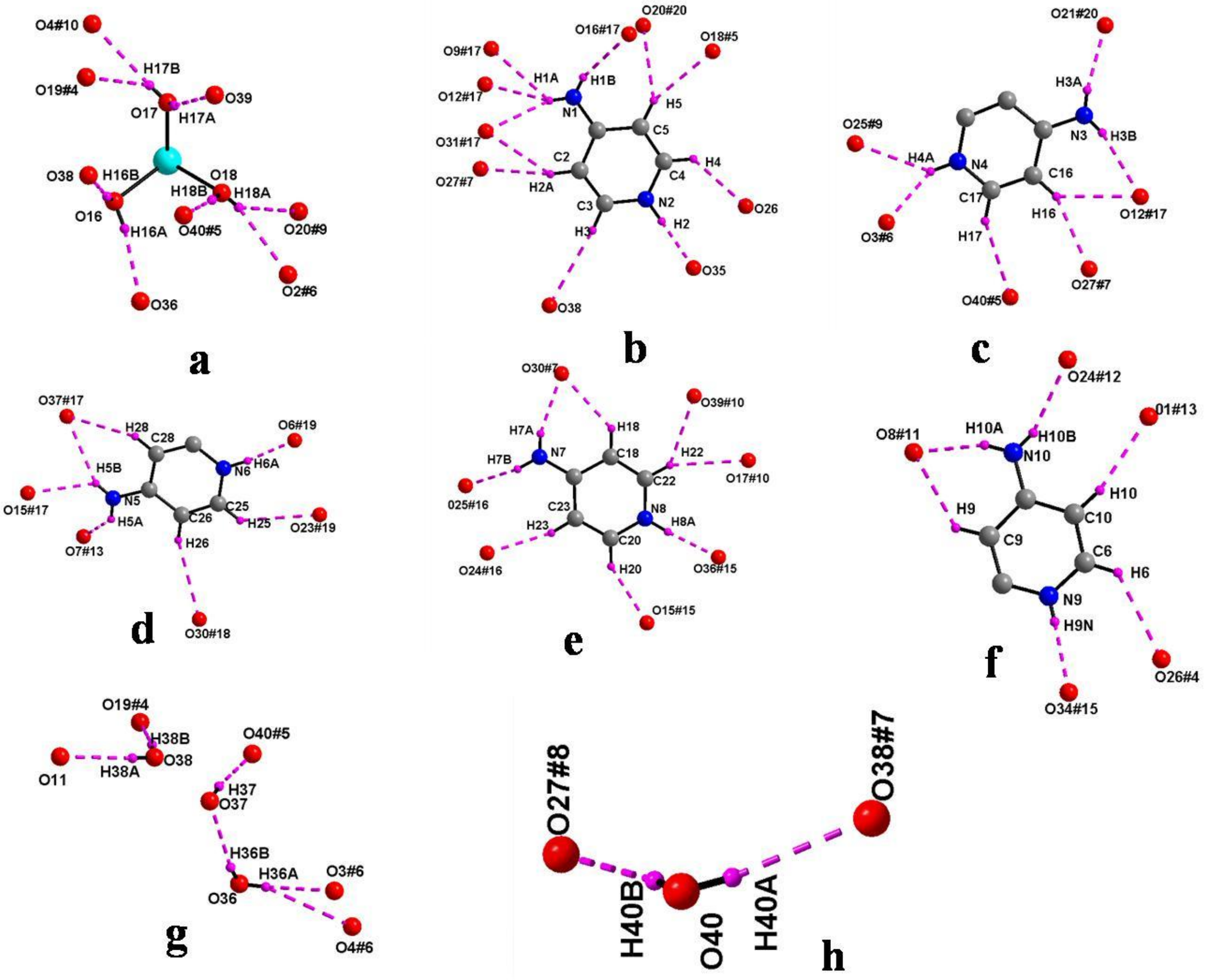

Supplementary Figure 7. Hydrogen bonding environment around (a) {Zn}, (b) {N1N2}, (c) {N3N4}, (d) {N5N6}, (e) {N7N8}, (f) {N9N10} and (g-h) water moieties in the crystals $[\{4\text{-ampH}\}_{10}\{Zn(H_2O)_6\}][V_{10}O_{28}]_2 \cdot 10H_2O$ (**7**). Symmetry codes: #1, -x,-y,-z; #2,-x+1,-y+1,-z; #3, -x,-y+1,-z; #4,x,-y+3/2,z-1/2; #5, x+1,y+1,z; #6, -x+1,y+1/2,-z+1/2; #7, -x+1,y+1/2,-z+3/2; #8, -x,y+1/2,-z+1/2;#9, x,y+1,z; #10, x,-y+3/2,z+1/2; #11,-x+1,y-1/2,-z+1/2; #12, x,-y+1/2,z+1/2; #13, x+1,y,z; #14, x-1,y,z; #15, -x+1,-y+1,-z+1; #16, x,y,z-1; #17, x,y,z+1; #18, x+1,-y+1/2,z+1/2; Color codes: O, red; C, grey; H, purple; N, blue.

1.2 Supplementary Tables

Table S1. Crystal data and structure refinement for compound **1**

Entry	**1**
Molecular formula	$CoH_{44}Na_4O_{52}V_{10}$
Formula weight	1536.64
Temperature (K)	298(2)
Wavelength (Å)	0.71073
Crystal system	Triclinic
Space group	*P*-1
a (Å)	8.9888(5)
b (Å)	11.2680 (17)
c (Å)	11.6587(15)
α (deg)	105.292(15)
β (deg)	97.570(2)
γ (deg)	100.77(3)
Volume ($Å^3$)	1098.4(3)
Z	1
ρ (g cm^{-3})	2.323
μ (mm^{-1})	2.567
F (000)	761
Crystal size (mm^3)	0.24x0.18x0.14
Θ range for data collection (º)	2.24 to 25.93
Reflections collected/unique	11396/4243
R(int)	0.0205
Data/restraints/parameters	4243/0/395
Goodness of fit on F^2	1.234
Final R indices [I > 2 sigma(I)]	0.0430,0.0969
R indices (all data)	0.0450/0.0977
Largest diff. Peak and hole (e.$Å^{-3}$)	1.418/-1.524

Table S2. Bond lengths [Å] and angles [˚] for decavanadate cluster of compound **1**

Bond Lengths (Å)

V(7)-O(16)	1.617(3)	V(7)-O(9)	1.800(3)
V(7)-O(11)	1.845(3)	V(7)-O(14)	1.965(3)
V(7)-O(17)	2.009(3)	V(7)-O(10)	2.238(3)
V(8)-O(18)	1.618(3)	V(8)-O(20)	1.791(3)
V(8)-O(19)	1.846(3)	V(8)-O(10)	2.237(3)
V(9)-O(15)	1.685(3)	V(9)-O(13)	1.687(3)
V(9)-O(14)	1.951(3)	V(9)-O(10)	2.098(3)
V(10)-O(8)	1.594(3)	V(10)-O(7)	1.827(3)
V(10)-O(19)	1.862(3)	V(10)-O(9)	1.890(3)
V(10)-O(13)	2.039(3)	V(10)-O(10)	2.296(3)
V(11)-O(12)	1.614(3)	V(11)-O(7)	1.822(3)
V(11)-O(11)	1.836(3)	V(11)-O(20)	1.899(3)
V(11)-O(10)	2.291(3)		

Bond angles [˚]

O(16)-V(7)-O(9)	103.89(15)	O(16)-V(7)-O(11)	101.89(14)
O(9)-V(7)-O(11)	94.39(14)	O(16)-V(7)-O(14)	100.54(14)
O(9)-V(7)-O(14)	91.39(13)	O(11)-V(7)-O(14)	154.73(12)
O(16)-V(7)-O(17)	99.41(14)	O(9)-V(7)-O(17)	155.36(13)

O(11)-V(7)-O(17)	88.53(13)	O(14)-V(7)-O(17)	76.42(12)
O(16)-V(7)-O(10)	174.48(14)	O(9)-V(7)-O(10)	80.91(12)
O(11)-V(7)-O(10)	80.24(12)	O(14)-V(7)-O(10)	76.41(11)
O(17)-V(7)-O(10)	75.48(11)		

Table S3. Hydrogen bond distances and angles for compound **1** [Å and º].

D–H···A	d(D–H)	d(H···A)	d(D···A)	<(DHA)
O(3)-H(3B)... O(18)	0.87(8)	2.00(8)	2.839(5)	162(7)
O(3)-H(3A)... O(29)	0.86(7)	1.87(7)	2.720(5)	172(6)
O(2)-H(18B)... O(20)#5	0.77(7)	1.93(7)	2.688(5)	168(6)
O(2)-H(18A)... O(28)#2	0.82(6)	1.97(6)	2.778(5)	169(5)
O(1)-H(1B)... O(19)#2	0.72(7)	1.98(7)	2.707(5)	178(7)
O(1)-H(1A)... O(12)#6	0.82(7)	1.95(7)	2.766(5)	172(6)
O(29)-H(29A)... O(15)#7	0.73(7)	2.07(7)	2.801(5)	178(7)
O(29)-H(29B)... O(17)#1	0.64(7)	2.27(7)	2.872(5)	159(8)
O(29)-H(29B)... O(11)#1	0.64(7)	2.64(7)	3.123(5)	135(8)
O(30)-H(30A)... O(25)	0.86(6)	2.03(6)	2.881(5)	169(5)
O(23)-H(23A)... O(29)#2	0.78(5)	2.11(5)	2.874(5)	169(5)
O(23)-H(23B)... O(14)#8	0.74(6)	2.06(6)	2.801(4)	176(6)

O(27)-H(27B)...O(16)#8	0.79(7)	2.07(7)	2.858(5)	174(7)
O(27)-H(27A)...O(9)#9	0.65(8)	2.55(8)	3.162(5)	159(9)
O(28)-H(28B)...O(19)	0.70(6)	2.23(6)	2.861(5)	152(6)
O(28)-H(28A)...O(18)#6	0.82(7)	2.03(7)	2.817(5)	159(6)
O(22)-H(22A)...O(26)#8	0.85(6)	1.95(6)	2.591(7)	131(5)
O(22)-H(22B)...O(12)#5	0.90(8)	2.07(8)	2.877(5)	150(7)
O(25)-H(25B)...O(9)#4	0.73(7)	2.00(7)	2.732(5)	180(8)
O(25)-H(25A)...O(16)#10	0.77(6)	2.16(6)	2.907(5)	165(5)
O(24)-H(24A)...O(11)#10	0.67(6)	2.23(7)	2.874(5)	162(7)
O(24)-H(24B)...O(13)#8	0.81(6)	2.22(6)	3.017(5)	171(5)

Symmetry transformations used to generate equivalent atoms: #1, -x+2,-y+1,-z+1; #2, -x+1,-y+1,-z ; #3, -x+1,-y,-z-1; #4, -x+1,-y,-z; #5, x-1,y,z; #6, -x+2,-y+1,-z; #7, -x+1,-y+1,-z+1; #8, x,y,z-1; #9, -x+2,-y,-z; #10, x-1,y,z-1;

Table S4. Hydrogen bonds and angles for compound **3** [Å and º].

D-H···A	d(D-H)	d(H···A)	d(D···A)	<(DHA)
C(4)-H(4A)... O(14)#4	0.97	2.81	3.705(16)	153.8
C(4)-H(4A)... O(12)#4	0.97	2.60	3.418(16)	141.6
C(3)-H(3B)... O(3)#5	0.97	2.53	3.450(16)	157.8
C(2)-H(2A)... O(12)#4	0.97	2.46	3.319(16)	147.1
C(2)-H(2B)... O(10)#6	0.97	2.40	3.286(17)	151.7
C(5)-H(5A)... O(10)#6	0.97	2.68	3.495(18)	141.5
C(1)-H(1B)... O(10)#6	0.97	2.51	3.358(16)	146.3
C(1)-H(1B)... O(13)#6	0.97	2.51	3.297(17)	138.6
C(1)-H(1A)... O(5)#2	0.97	2.45	3.381(16)	160.0
C(1)-H(1A)... O(7)#2	0.97	2.77	3.528(17)	136.0
C(3)-H(3A)... O(3)#2	0.97	2.31	3.273(16)	170.1
C(6)-H(6B)... O(4)#2	0.97	2.56	3.501(17)	164.6
C(2)-H(2A)... O(2)#5	0.97	2.45	3.263(16)	140.7
C(3)-H(3B)... O(2)#5	0.97	2.72	3.453(17)	132.8
C(4)-H(4B)... O(11)#7	0.97	2.62	3.542(17)	159.5
C(5)-H(5B)... O(1)#8	0.97	2.45	3.359(17)	156.7

Symmetry transformations used to generate equivalent atoms:

#1, -x+1,-y+1,-z; #2, x,-y+3/2,z+1/2; #3, x,-y+3/2,z-1/2; #4, x+1,-y+3/2,z+3/2; #5, -x+2,y+1/2,-z+3/2; #6, x,y,z+1; #7, x+1,y,z+1; #8, -x+2,-y+1,-z+1; **Table S5.**

Hydrogen bond distance and angles for compound **4** [Å and °]

D–H···A	d(D–H)	d(H···A)	d(D···A)	<(DHA)
O(20)-H(20B)... O(6)#2	0.86(9)	2.01(9)	2.846(6)	164(8)
O(22)-H(22B)... O(13)#3	0.92(8)	1.85(8)	2.767(5)	171(7)
O(19)-H(19B)...O(12)#3	0.80(8)	1.93(8)	2.705(5)	162(8)
C(2)-H(2)... O(20)#4	0.93(8)	2.53(8)	3.272(8)	138(6)
N(2)-H(2A)... O(14)#5	0.82(8)	2.27(8)	3.056(8)	162(7)
O(17)-H(17B)... O(9)	0.82(7)	1.95(7)	2.750(5)	166(6)
N(2)-H(2B)... O(3)	0.66(8)	2.37(8)	2.991(7)	157(8)
O(17)-H(17B)... O(9)	0.82(7)	1.95(7)	2.750(5)	166(6)
O(15)-H(15B)... O(20)	0.59(6)	2.24(6)	2.804(8)	163(8)
O(15)-H(15A) ...O(6)	0.91(10)	1.92(10)	2.816(5)	169(8)
O(18)-H(18A) ...O(11)#6	0.54(8)	2.13(8)	2.650(5)	160(12)
O(17)-H(17A)... O(10)#6	0.56(6)	2.26(6)	2.811(6)	174(9)
O(17)-H(17B)... O(9)	0.82(7)	1.95(7)	2.750(5)	166(6)
C(7)-H(7)... O(10)#6	0.96(9)	2.37(9)	3.307(8)	166(7)
C(7)-H(7)... O(8)#6	0.96(9)	2.88(8)	3.301(8)	108(6)

C(10)-H(10)...O(21)#7	1.01(6)	2.88(7)	3.745(13)	144(5)
N(3)-H(3N)...O(7)#8	0.90(12)	1.77(12)	2.662(5)	172(11)
C(9)-H(9)...O(14)#5	1.04(10)	2.49(10)	3.437(9)	152(7)

Symmetry transformations used to generate equivalent atoms:

#1 -x+1,-y+2,-z+2; #2 -x+1,-y+1,-z+2; #3 x,y-1,z; #4 -x+1,-y+1,-z+1; #5 -x+1,-y+2,-z+1 #6 - x+2,-y+2,-z+2; #7 -x+2,-y+1,-z+1; #8, x,y,z-1.

Table S6. Hydrogen bonds and angles for compound **5** [Å and º].

D–H···A	d(D–H)	d(H···A)	d(D···A)	<(DHA)
N(2)-H(2N)...O(36)#4	0.69(5)	2.12(5)	2.801(5)	167(5)
C(4)-H(4)...O(12)#5	0.89(5)	2.33(5)	3.123(5)	148(5)
C(5)-H(5)...O(27)	0.87(5)	2.30(5)	3.161(5)	168(5)
N(1)-H(1A)...O(25)	0.79(5)	2.29(5)	3.036(4)	156(5)
N(1)-H(1B)...O(6)#6	0.88(5)	2.05(5)	2.897(4)	162(4)
C(2)-H(2)...O(6)#6	0.84(4)	2.56(4)	3.245(4)	140(3)
C(3)-H(3)...O(31)#7	0.89(4)	2.62(4)	3.333(6)	138(4)
N(3)-H(3A)...O(33)#8	0.59(5)	2.65(5)	3.177(5)	150(6)
N(3)-H(3B)...O(13)#9	0.85(4)	2.36(4)	3.166(6)	158(4)

C(6)-H(6)...O(14)#9	0.87(4)	2.58(4)	3.354(4)	148(3)
N(4)-H(4N)...O(4)#8	0.79(4)	1.91(4)	2.702(4)	174(4)
C(8)-H(8)...O(1)#8	0.88(4)	2.60(4)	3.264(4)	133(4)
C(9)-H(9)...O(35)#10	0.76(4)	2.75(4)	3.471(5)	159(4)
C(15)-H(15)...O(26)#10	0.85(4)	2.50(4)	3.328(4)	164(4)
N(5)-H(5A)...O(27)#10	0.79(4)	2.24(5)	2.976(4)	154(4)
N(5)-H(5B)...O(7)#6	0.87(5)	2.13(5)	2.933(4)	155(4)
C(12)-H(12)...O(7)#6	0.92(5)	2.62(5)	3.336(5)	135(4)
N(6)-H(6N)...O(2)#8	0.80(6)	1.91(6)	2.685(4)	163(6)
C(14)-H(14)...O(1)#8	0.92(4)	2.64(4)	3.352(4)	135(3)
N(7)-H(7A)...O(20)	0.76(4)	2.12(5)	2.844(4)	162(4)
N(7)-H(17B)...O(9)#6	0.81(5)	2.31(5)	3.033(4)	150(5)
C(17)-H(17)...O(9)#6	0.93(5)	2.63(5)	3.347(4)	134(4)
C(17)-H(17)...O(14)#6	0.93(5)	2.41(5)	3.268(4)	154(4)
C(18)-H(18)...O(29)#11	0.87(4)	2.76(4)	3.477(6)	140(4)
N(8)-H(8N)...O(16)#12	0.85(5)	2.05(5)	2.855(4)	159(5)
N(8)-H(8N)...O(25)#11	0.85(5)	2.70(5)	3.319(4)	131(4)
N(10)-H(10N)...O(21)#2	0.72(5)	1.97(5)	2.677(4)	168(5)
C(23)-H(23)...O(30)#11	0.91(5)	2.81(5)	3.516(5)	135(4)
N(9)-H(9A)...O(30)#11	0.65(7)	2.63(7)	3.216(9)	152(9)
N(9)-H(9A)...O(12)#13	0.65(7)	2.43(7)	2.934(5)	136(9)

N(9)-H(9B)...O(32)#11	0.93(7)	2.06(7)	2.937(5)	159(5)
O(33)-H(33B)... O(28)#14	0.63(4)	2.18(4)	2.807(4)	177(5)
O(33)-H(33A)... O(36)#15	0.88(4)	1.92(4)	2.795(4)	173(3)
O(35)-H(35A)... O(29)	0.53(4)	2.32(4)	2.829(5)	160(7)
O(34)-H(34B)... O(18)#2	0.61(6)	2.41(6)	2.967(4)	153(7)
O(34)-H(34A)... O(31)#16	0.71(5)	2.13(5)	2.790(6)	156(6)
O(28)-H(28A)... O(8)#6	0.80(5)	2.11(6)	2.906(4)	174(5)
O(28)-H(28B)... O(24)	0.72(5)	1.97(5)	2.686(4)	170(5)
O(36)-H(36A)... O(16)#17	0.82(5)	2.06(5)	2.824(4)	154(5)
O(36)-H(36B)... O(30)#15	0.61(6)	2.27(6)	2.841(5)	157(8)
O(36)-H(36B)... O(12)#18	0.61(6)	2.82(6)	3.254(4)	132(7)
O(29)-H(29A)... O(14)#9	0.71(5)	2.04(5)	2.746(4)	170(5)
O(29)-H(29B)... O(28)#14	0.72(5)	2.15(5)	2.815(5)	154(5)
O(30)-H(30B)... O(29)	0.82(6)	2.06(6)	2.866(5)	169(5)
O(30)-H(30A)... O(10)#8	0.87(5)	1.98(5)	2.837(4)	168(4)
O(31)-H(31B)... O(27)#15	0.73(6)	2.17(6)	2.903(5)	176(6)
O(31)-H(31A)... O(5)#17	0.61(5)	2.37(5)	2.976(5)	170(7)

Symmetry transformations used to generate equivalent atoms:

#1 -x,-y,-z #2 -x+1,-y+1,-z #3 -x,-y+1,-z; #4 x,-y+3/2,z-1/2; #5 x+1,y+1,z; #6, -x+1,y+1/2,-z+1/2; #7, -x+1,y+1/2,-z+3/2; #8, -x,y+1/2,-z+1/2; #9, x,y+1,z; #10, x,-y+3/2,z+1/2; #11, -

x+1,y-1/2,-z+1/2; #12, x,-y+1/2,z+1/2; #13, x+1,y,z; #14, x-1,y,z; #15, -x+1,-y+1,-z+1; #16, x,y,z-1; #17, x,y,z+1; #18, x+1,-y+1/2,z+1/2.

Table S7. Hydrogen bonds and angles for compound **6** [Å and º].

D-H···A	d(D–H)	d(H···A)	d(D···A)	<(DHA)
O(42)-H(42A)···O(3)#4	0.58(8)	2.46(8)	2.987(6)	154(10)
O(42)-H(42A)···O(4)#4	0.58(8)	2.53(8)	2.958(6)	134(10)
O(42)-H(42B)···O(44A)	0.70(8)	1.83(8)	2.481(12)	155(8)
O(42)-H(42B)···O(44)	0.70(8)	2.17(8)	2.849(8)	165(8)
O(35)-H(35A)···O(4)	0.68(7)	2.89(7)	3.341(5)	126(6)
O(35)-H(35A)···O(42)#5	0.68(7)	2.25(7)	2.900(6)	160(7)
O(35)-H(35B)···O(39)	0.87(6)	1.84(6)	2.697(5)	168(5)
O(39)-H(39A)···O(16)#5	0.73(6)	2.03(7)	2.753(5)	173(7)
O(39)-H(39B)···O(21)	0.84(7)	1.88(8)	2.701(5)	167(7)
O(40)-H(40B)···O(20)#6	0.79(8)	2.34(8)	2.989(6)	140(7)
O(40)-H(40A)···O(4)	0.86(8)	1.93(8)	2.761(5)	163(7)
O(41)-H(41A)···O(10)#7	0.87(7)	2.23(6)	2.941(5)	140(5)
O(41)-H(41A)···O(13)#7	0.87(7)	2.12(6)	2.743(5)	128(5)

O(41)-H(41B)···O(11)	0.67(8)	2.09(8)	2.710(5)	153(9)
O(38)-H(38B)···O(20)#8	0.48(8)	2.32(9)	2.777(6)	158(14)
O(38)-H(38A)···O(17)#6	0.84(7)	1.86(7)	2.685(6)	167(6)
O(36)-H(36B)···O(44A)	0.71(6)	2.14(6)	2.812(13)	160(6)
O(36)-H(36B)···O(44)	0.71(6)	2.09(6)	2.788(6)	171(6)
O(36)-H(36A)···O(25)	0.68(7)	2.11(7)	2.754(5)	158(8)
O(37)-H(37A)···O(35)#4	0.79(7)	1.95(8)	2.722(5)	165(7)
O(37)-H(37B)···O(22)#4	0.90(10)	2.16(10)	3.030(5)	163(8)
O(37)-H(37B)···O(43A)#4	0.90(10)	2.12(9)	2.700(12)	122(7)
C(13)-H(13)···O(29)	0.83(5)	2.76(5)	3.416(6)	137(4)
C(13)-H(13)···O(31)	0.83(5)	2.73(6)	3.462(7)	147(5)
C(15)-H(15)···O(33)#4	1.07(7)	2.57(7)	3.518(8)	148(5)
C(15)-H(15)···O(34)#4	1.07(7)	2.67(7)	3.526(8)	136(5)
C(6)-H(6)···O(18)#6	0.86(7)	2.54(8)	3.228(6)	138(6)
N(4)-H(4B)···O(19)#6	0.71(6)	2.39(6)	2.869(6)	126(6)
N(4)-H(4A)···O(40)	0.90(9)	2.05(9)	2.884(8)	155(8)
C(9)-H(9)···O(5)	0.82(6)	2.69(6)	3.411(6)	147(5)
C(8)-H(8)···O(6)	0.92(5)	2.49(5)	3.192(6)	134(4)
N(3)-H(3N)···O(12)#4	0.83(6)	1.87(6)	2.704(5)	175(6)
C(7)-H(7)···O(8)#4	0.95(6)	2.67(6)	3.392(6)	134(4)

C(7)-H(7)···O(3)#4	0.95(6)	2.63(6)	3.305(6)	129(4)
C(4)-H(4)···O(42)#9	0.92(7)	2.61(7)	3.377(7)	141(5)
N(1)-H(1N)···O(43)#9	0.87(7)	2.83(7)	3.390(7)	124(5)
N(2)-H(2B)···O(1)#3	0.75(9)	2.52(9)	3.189(6)	151(9)
C(1)-H(1)···O(14)#4	0.81(6)	2.58(6)	3.331(6)	155(5)
N(2)-H(2A)···O(7)#9	0.88(5)	2.01(6)	2.875(6)	171(5)
N(2)-H(2B)···O(6)	0.75(9)	2.48(9)	3.094(6)	141(9)
C(3)-H(3)···O(1)#3	0.88(6)	2.48(6)	3.243(6)	145(5)
C(4)-H(4)···O(24)#9	0.92(7)	2.39(7)	3.026(6)	127(5)
N(1)-H(1N)···O(24)#9	0.87(7)	2.43(7)	3.018(6)	126(5)
N(1)-H(1N)···O(22)#9	0.87(7)	2.18(7)	2.952(6)	147(6)
N(1)-H(1N)···O(43A)#9	0.87(7)	2.88(7)	3.444(12)	124(5)

Symmetry transformations used to generate equivalent atoms:

#1, -x+1, -y+2, -z; #2, -x+2, -y+1, -z #3, -x+1, -y, -z+1 #4, x, y+1, z; #5, x, y-1, z #6 x-1,y,z #7 -x,-y,-z+1 #8 x-1,y+1,z #9 -x+1,-y+1,-z+1.

Table S8. Hydrogen bond distances and angles for compound **7** [Å and °].

D–H···A	d(D–H)	d(H···A)	d(D···A)	<(DHA)
O(38)-H(38B)... O(19)#4	0.69(6)	2.01(6)	2.683(5)	168(7)
O(38)-H(38A)... O(11)	0.69(5)	2.21(6)	2.902(5)	176(6)
O(37)-H(37)...O(40)#5	0.74(7)	2.12(7)	2.853(6)	171(8)
O(36)-H(36B)... O(37)	0.70(5)	2.16(5)	2.837(6)	163(6)
O(36)-H(36A)... O(3)#6	0.74(5)	2.11(5)	2.828(5)	165(5)
O(36)-H(36A)... O(4)#6	0.74(5)	2.79(5)	3.235(5)	121(4)
O(40)-H(40A)... O(38)#7	0.74(6)	2.11(6)	2.788(6)	151(6)
O(40)-H(40B)... O(27)#8	0.73(6)	2.02(6)	2.746(5)	171(6)
O(18)-H(18B)... O(40)#5	0.81(6)	2.00(6)	2.804(6)	176(6)
O(18)-H(18A)... O(20)#9	0.65(5)	2.59(6)	3.113(6)	139(6)
O(18)-H(18A)... O(2)#6	0.65(5)	2.30(6)	2.852(5)	144(6)
O(16)-H(16A)... O(36)	0.79(5)	2.01(5)	2.793(5)	171(5)
O(16)-H(16B)... O(38)	0.66(5)	2.13(5)	2.795(6)	175(6)

O(17)-H(17B)...O(19)#4	0.68(6)	2.53(7)	2.981(5)	127(7)
O(17)-H(17B)...O(4)#10	0.68(6)	2.30(7)	2.963(5)	165(7)
O(17)-H(17A)...O(39)	0.73(6)	2.12(6)	2.806(6)	158(6)
C(9)-H(9)...O(8)#11	0.95	2.61	3.328(6)	132.6
N(10)-H(10A)...O(8)#11	0.83(7)	2.13(7)	2.910(6)	157(6)
N(10)-H(10B)...O(24)#12	0.76(5)	2.28(5)	2.976(6)	152(5)
C(10)-H(10)...O(1)#13	0.95	2.40	3.327(5)	166.3
C(6)-H(6)...O(26)#14	0.95	2.63	3.344(5)	132.2
N(9)-H(9N)...O(34)#15	0.73(5)	1.96(5)	2.684(5)	176(5)
C(22)-H(22)...O(39)#10	0.95	2.56	3.332(7)	138.3
C(22)-H(22)...O(17)#10	0.95	2.76	3.645(6)	154.6
N(8)-H(8A)...O(36)#15	0.88	1.93	2.795(5)	169.4
C(20)-H(20)...O(15)#15	0.95	2.31	3.107(6)	141.4
C(23)-H(23)...O(24)#16	0.95	2.25	3.159(6)	158.6
N(7)-H(7B)...O(25)#16	0.87(6)	2.17(6)	3.002(5)	161(5)
N(7)-H(7A)...O(30)#7	0.78(5)	2.14(5)	2.892(5)	163(5)
C(18)-H(18)...O(30)#7	0.95	2.47	3.223(5)	135.9
C(28)-H(28)...O(37)#17	0.95	2.82	3.523(7)	131.3
N(5)-H(5B)...O(37)#17	0.78(8)	2.47(8)	3.176(10)	150(8)
N(5)-H(5B)...O(15)#17	0.78(8)	2.34(8)	2.924(6)	132(8)
N(5)-H(5A)...O(7)#13	0.81(6)	2.33(6)	2.935(6)	133(5)

C(26)-H(26)...O(30)#18	0.95	2.82	3.700(6)	154.3
C(25)-H(25)...O(23)#19	0.95	2.83	3.532(5)	131.2
N(6)-H(6A)...O(6)#19	0.88	1.79	2.670(4)	173.8
N(4)-H(4A)...O(25)#9	0.88	2.63	3.331(5)	137.4
N(4)-H(4A)...O(3)#6	0.88	2.04	2.846(5)	151.3
C(17)-H(17)...O(40)#5	0.95	2.65	3.466(7)	143.7
C(16)-H(16)...O(27)#7	0.95	2.39	3.263(5)	153.1
N(3)-H(3B)...O(12)#17	0.76(5)	2.36(5)	3.020(5)	146(5)
N(3)-H(3A)...O(21)#20	0.77(5)	2.10(5)	2.833(5)	161(5)
C(5)-H(5)...O(20)#20	0.95	2.77	3.474(5)	131.3
C(5)-H(5)...O(18)#5	0.95	2.54	3.428(6)	155.2
C(4)-H(4)...O(26)	0.95	2.56	3.248(5)	129.2
N(2)-H(2)...O(35)	0.88	1.82	2.697(4)	174.6
C(3)-H(3)...O(38)	0.95	2.81	3.756(6)	173.5
C(2)-H(2A)...O(27)#7	0.95	2.50	3.348(5)	148.4
C(2)-H(2A)...O(31)#17	0.95	2.81	3.535(5)	134.1
N(1)-H(1A)...O(31)#17	0.79(5)	2.42(5)	3.158(6)	156(4)
N(1)-H(1A)...O(12)#17	0.79(5)	2.48(5)	3.111(5)	138(4)
N(1)-H(1A)...O(9)#17	0.79(5)	2.74(5)	3.287(5)	128(4)
N(1)-H(1B)...O(16)#17	0.73(6)	2.45(6)	3.128(6)	154(6)

Symmetry transformations used to generate equivalent atoms:

#1, -x+1,-y,-z+1; #2, -x,-y+2,-z+1; #3, -x+1,-y,-z; #4, x+1,y-1,z; #5, x,-y+1/2,z-1/2; #6, x,y-1,z;#7, x,-y+1/2,z+1/2; #8, x,y,z+1; #9, -x,-y+1,-z+1; #10, -x+1,-y+1,-z+1; #11, x+1,y+1,z; #12, x+1,-y+3/2,z-1/2; #13, -x+1,y-1/2,-z+1/2; #14, -x+1,-y+1,-z; #15, x,y+1,z; #16, x,-y+3/2,z-1/2; #17, -x+1,y+1/2,-z+1/2; #18, x+1,-y+1/2,z+1/2; #19, x+1,y,z; #20, -x,y-1/2,-z+1/2.

#################End of the Supplementary Information#######################
